# Processing genome-wide association studies within a repository of heterogeneous genomic datasets

**DOI:** 10.1186/s12863-023-01111-y

**Published:** 2023-03-03

**Authors:** Anna Bernasconi, Arif Canakoglu, Federico Comolli

**Affiliations:** Department of Electronics, Information and Bioengineering, Politecnico di Milano, Via Ponzio 34/5, 20133 Milano, Italy

**Keywords:** Data integration, Processed datasets, Tertiary data analysis, Genomics, Multiomics studies, GWAS

## Abstract

**Background:**

Genome Wide Association Studies (GWAS) are based on the observation of genome-wide sets of genetic variants – typically single-nucleotide polymorphisms (SNPs) – in different individuals that are associated with phenotypic traits. Research efforts have so far been directed to improving GWAS techniques rather than on making the results of GWAS interoperable with other genomic signals; this is currently hindered by the use of heterogeneous formats and uncoordinated experiment descriptions.

**Results:**

To practically facilitate integrative use, we propose to include GWAS datasets within the META-BASE repository, exploiting an integration pipeline previously studied for other genomic datasets that includes several heterogeneous data types in the same format, queryable from the same systems. We represent GWAS SNPs and metadata by means of the Genomic Data Model and include metadata within a relational representation by extending the Genomic Conceptual Model with a dedicated view. To further reduce the gap with the descriptions of other signals in the repository of genomic datasets, we perform a semantic annotation of phenotypic traits. Our pipeline is demonstrated using two important data sources, initially organized according to different data models: the NHGRI-EBI GWAS Catalog and FinnGen (University of Helsinki). The integration effort finally allows us to use these datasets within multi-sample processing queries that respond to important biological questions. These are then made usable for multi-omic studies together with, e.g., somatic and reference mutation data, genomic annotations, epigenetic signals.

**Conclusions:**

As a result of the our work on GWAS datasets, we enable 1) their interoperable use with several other homogenized and processed genomic datasets in the context of the META-BASE repository; 2) their big data processing by means of the GenoMetric Query Language and associated system. Future large-scale tertiary data analysis may extensively benefit from the addition of GWAS results to inform several different downstream analysis workflows.

**Supplementary Information:**

The online version contains supplementary material available at 10.1186/s12863-023-01111-y.

## Background

Genome-wide association studies (GWAS) aim to find statistical associations between genetic variants and traits of interest using data from a large number of individuals [1, 2]. They have brought a revolution to the study of genetics and complex diseases, identifying more than 50k associations between variants – typically single-nucleotide polymorphisms (SNPs) – and complex traits and diseases. These results are used to augment predictions for a variety of human “phenotypes”, an umbrella term that includes a large range of semantically distinct concepts such as traits, diseases, medical signs, and symptoms (e.g., body mass index, hair color, type 2 diabetes, and Alzheimer’s disease [3, 4]).

Several data sources provide open access to limited amounts of summary-level GWAS, including the GWAS Atlas [5] (with a wide range of species), GWASdb v2 [6] (offline as of May 26th, 2022), GWAS Central [7] (a toolkit for integrative access), the Open Access Database of Genome-wide Association Results [8], and PheGenI (GWASs with NCBI databases such as Gene, dbGap, and OMIM). Other resources are only available for specific phenotypes (such as the Amyotrophic Lateral Sclerosis online Database [9]), for specific species (such as the AraGWAS Catalog [10]), or for specific purposes (such as DistiLD [11], checking the linkage disequilibrium blocks onto which SNPs and genes are mapped).

In this work, we focus on human GWAS, in particular from the NHGRI-EBI GWAS Catalog [12] and FinnGen [13]. GWAS Catalog is a collection of published genome-wide association studies that enable investigations to identify causal variants, understand disease mechanisms, and establish targets for novel therapies. A team of curators manually add metadata about publication, study design, sample, and trait information. Many information from GTEx [14] are also integrated. The FinnGen project [13] was launched in Finland in 2017, to collect biological samples from 500K participants (about 10% of the overall Finland population) in a span of time of six years with the aim of informing diagnostics and new therapies through genetic research. The University of Helsinki is responsible for the study, to which the nation-wide network of Finnish biobanks participates, having the Helsinki Biobank coordinating the sample collection.

Currently, the several mentioned efforts are directed to systematize and enrich the quantity of knowledge available for GWAS, with attempts to 1) homogenize the use of different ontologies that describe phenotypic observations across databases [7]; 2) make GWAS summary statistics more and more FAIR [15]. All efforts conducted thus far appear to be focusing on GWAS as a data type that is isolated, or at most paired with annotations [16]. Unfortunately, data from different sources and types are typically made available using different protocols, expressed using heterogeneous data models and formats, hampering the inter-operation of GWAS information with other (epi)genomic signals. To address the lack of solutions for integrating GWAS with diverse genome-related datasets in a unique format, we propose to include GWAS summary-level datasets within a repository by adopting a set of models and frameworks that have been previously applied successfully. First, we model GWAS datasets using the Genomic Data Model (GDM [17]), which explicitly separates region data (sets of regions described by a chromosome number, start-stop coordinates and other attributes) from metadata (experiment descriptions). Then, we use and extend the Genomic Conceptual Model (GCM [18]) for representing the descriptions of GWAS datasets, allowing to correctly locate them in the context of large databases. We process GWAS datasets within the structured integration META-BASE framework [19], downloading them from arbitrary sources (in this article we consider GWAS Catalog and FinnGen), transforming them into the desired GDM-based format, mapping the relevant information within a GCM-based relational database, where a semantic enrichment is performed to link phenotypes to recommended or user-specified ontologies. The potential of our integration approach is finally illustrated by means of four biologically-relevant queries with the GenoMetric Query Language (GMQL [20]), operating upon aligned Next Generation Sequencing genomic data from a variety of data sources. GMQL provides parallel computation in the cloud [21], supporting queries over thousands of samples at the same time, taking into account region-relative positions and distances.

## Methods

**Fig. 1:**
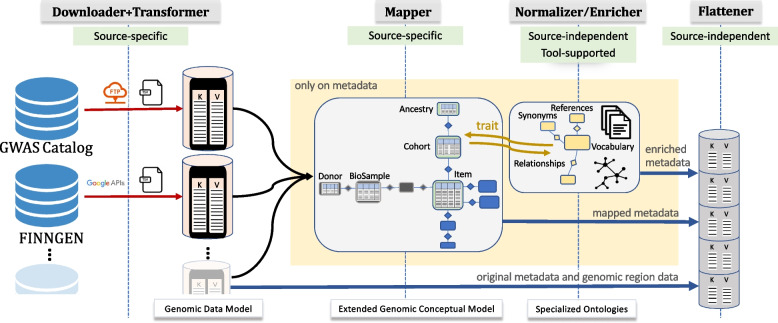
Data extraction and integration pipeline including *Download*, *Transform* (based on the Genomic Data Model), *Map* (based on an extended version of the Genomic Conceptual Model), *Enrich*, and *Flatten* steps

We employ a structured data integration process, which allows to retrieve GWAS datasets from their sources and import them in our systems. The pipeline is summarized in Fig. 1, representing the GWAS-specific instance of the META-BASE framework [19] of the GeCo project [22]^1^. The original META-BASE pipeline has been extended with specific modules for handling GWAS sources. The *Downloader* module has the ability to integrate relevant GWAS sources (here we restrict to two example sources). The *Transformer* module transforms data into a shared format – employing the Genomic Data Model on which the output format is based. The two following modules only act on metadata, leaving genomic region data unchanged: the *Mapper* module is in charge of the extraction of selected information and its representation within an extended version of the Genomic Conceptual Model, where heterogeneity is addressed at the schema level; the *Enricher* is in charge of the integration at the value semantics level. Finally, the *Flattener* is reused *as is* to handle the conversion back to the original file-based representation for processing within the GMQL system [21].

The execution of the overall process, composed of the five mentioned steps, is driven by an XML configuration file, selecting which phase to execute, URLs of API/FTP servers through which data are downloaded, local paths to the source-specific classes, and the local path to reach the source-specific GDM schemata (a list of fields and their data types).

### Download

The Downloader module connects to the endpoints of selected genomic data sources and produces files - both for the genomic data and its metadata - in original source-specific format, at the processing site hosting our repository. We build a collection of protocol-specific modules with few parameters to adapt them to new sources; tunings for each specific source may be necessary. For the scope of this project, we focused on two sources, detailed next.

The GWAS Catalog [12] started in 2015 within a collaborative project between EMBL-EBI (European Bioinformatics Institute) and NHGRI (National Human Genome Research Institute). New studies are found through weekly PubMed searches and new data are manually extracted from literature by domain experts, leveraging an automatic pipeline that annotates SNPs with external knowledge. Phenotypic traits are mapped to the Experimental Factor Ontology (EFO [24]). The repository of summary statistics contains three tab-separated files Ancestry, Studies and Associations, which can be downloaded from the dedicated FTP server (https://ftp.ebi.ac.uk). New versions of the repository are released monthly. In this work, we focus on the stable release of May 6th, 2021 that includes 16,854 studies, corresponding to 257,352 associations between SNPs and related traits. The Studies file contains one entry for each trait analysed in a study on PubMed; studies regarding multiple traits are split in multiple entries. The Ancestry file contains information about the cohorts of patients who participated to the studies (including cohort’s size and geographical provenance). The Associations file contains one row for each association (i.e., relation between an SNP and the study-targeted trait), equipped with statistical properties about the found correlations (e.g., *p*-value). The three files can be merged by means of a number of shared attributes, including the ‘STUDY ACCESSION’. Additional file 1 reports the complete list of the attributes of the Catalog with their description and indication of which file contains them as well as three tables with example content.

The FinnGen project [13] was born from the collaboration between private and public Finnish institutes, started in Autumn 2017. It aims to improve human health through genetic research, paving the road to personalized medicine with ad-hoc treatments. The project aims to reach a cohort of 500,000 participants by 2023: every Finnish person can join the project and become part of study cohorts by giving appropriate consent. All individuals are genotyped using GWAS. The outcome of these studies are the SNPs found relevant for the phenotypes under consideration, called ‘endpoints’ in the FinnGen context. Data can be accessed through different channels, both programmatically or via a web browser. The repository is updated twice a year and it becomes publicly available one year after it is produced. For our purposes, we consider the release 5, published in May 2021, containing the SNPs associated to 2,804 endpoints. The repository is composed by two main modules: summary statistic (including all the SNPs associated to the relative phenotype and statistical properties of the SNPs) and fine-mapping, not considered here (including the outcomes of the fine-mapping process with the SuSiE [25] and FINEMAP [26] softwares). In Additional file 2, we report the complete list of the attributes of the FinnGen summary statistics and three tables with example content. We download the manifest (made available for each project’s release), which contains a list of endpoints, one for each considered trait. We then call each trait endpoint, downloading the corresponding summary statistics file and saving all of them in the specified local folder.

### Transformation

The Transformer deals with the lack of agreement towards a standard data unit for genomic tertiary analysis. We propose to use the “sample” of the Genomic Data Model (GDM [17]), in contrast with other more complex or hierarchical solutions. The module takes, as its input, the data and metadata files resulting from the Download phase and transforms them into a GDM-compliant format, resolving two kinds of heterogeneity of genomic files: 1) the different data units; 2) the different data schemata within each unit. GDM is based on the notions of *datasets* and *samples*; datasets are collections of samples. Samples are the basic unit of information, containing experimental data that corresponds to a given individual and preparation (e.g., cell line and antibody used) that first undergoes sequencing (producing “raw data”), then alignment and calling steps (producing “processed data”). Each sample includes DNA segments or regions (called *region data*) and it is associated with information about the performed experiment, i.e., *metadata* describing the general properties of the sample. Genomic region and feature data can describe many molecular aspects, which are measured individually; the resulting variety of formats hampers their integration and comprehensive assessment. GDM provides a schema to the genomic features of DNA/RNA regions, making heterogeneous data self-describing and interoperable.

The original files are translated into the GDM format, which has a fixed part – representing the genomic coordinates – that guarantees the comparability of regions produced by different kinds of processing, and a variable part, i.e., data-type-specific attributes, describing region properties, reflecting the process of feature calling that produced the regions with their features specific of the particular processing experiment. GDM represents metadata using a free arbitrary semi-structured attribute-value pairs structure.

**Fig. 2:**
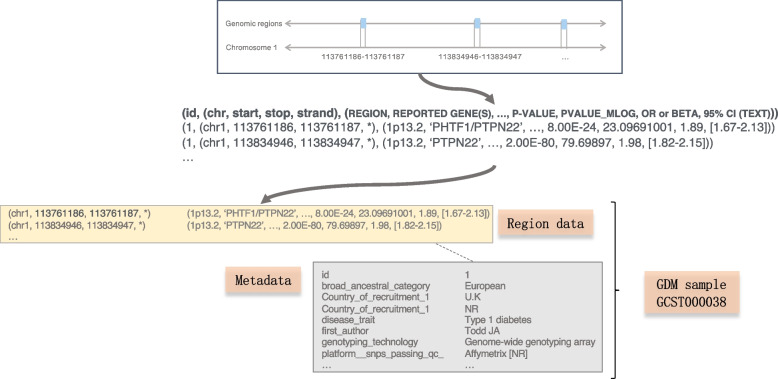
The Genomic Data Model [17] applied to GWAS data

Figure 2 shows how a GWAS data genome track is modeled as a GDM sample. Each blue rectangle becomes a region following the schema indicated in bold, where id is unique for each sample, chr, start, stop, strand are the fixed part, and REGION, REPORTED GENE(S), etc. are the variable one. Each region data file is tightly linked to its metadata file (with the same identifier). A typical GDM dataset (including the GWASs ones) contains thousands of samples like the one represented here. For GWAS Catalog, the transformation of the region data concerns only the four coordinate attributes; other attributes in the schema are reported as they are in Associations. The attribute chrom is derived from the original attribute CHR_ID when present, else from STRONGEST SNP-RISK ALLELE. The start is derived from CHR_POS, and end is the start+1. The strand information is not available, thus is set to ‘*’ by default. For FinnGen, the transformation phase only concerns coordinate attributes: chrom is called ‘#chrom’; start is derived from ‘pos’ and end is start+1 as we only represent SNPs; the strand is unknown (set to ‘*’). The remaining attributes in the schema are copied *as is*. Additional file 3 shows the correspondences between the region attributes of the two genomic sources GWAS Catalog and FinnGen.

### Mapping

The Mapper module is in charge of the integration at the schema-level of a set of transformed metadata produced for each source. The method applies local-to-global mappings using a syntax inspired to Datalog [27]. Mapping rules build relational rows from the key-value pairs output by the Transformer step to achieve the integration of different local schemata into a unique local one, i.e., an extended GWAS-compliant version of the Genomic Conceptual Model [28].

For supporting metadata search in a semantic-aware way, the Genomic Conceptual Model (GCM [18]) was previously proposed. The GCM is centered on the concept of item (i.e., typically a sample file containing genomic regions and their properties), described by four metadata views that explain its characteristics from the biological, technical, organizational, and computational perspectives. We map the concept of “study” on the existing Item entity; a GWAS “publication” maps to the GCM CaseStudy entity; a “trait” has a strong correspondence with the GCM BioSample’s disease attribute. Samples of typical sources integrated within the GCM are assigned to single individuals; for each biological sample we can retrieve the information about the donor(s) who provided it. However, GWASs are based on *cohorts of patients*, so the considered granularity is coarser with respect to already integrated datasets. The existing GCM biological view cannot capture the concept of “cohort” (a population that is divided into case and control individuals, either exhibiting or not a certain trait) and “ancestry” (of donor individuals) that are relevant for GWAS. For each GWAS sample (corresponding to summary statistics) we only know the cohort size and limited ancestral information, while detailed information about each single component of the cohort is not available. To meet the constraints of the considered class of studies, we have extended the GCM introducing a new GWAS-specific view; the resulting schema, called Extended Genomic Conceptual Model, can be appreciated in [28].

According to this view, the central entity Item is interpreted as a GWAS study, i.e., a file that contains all the SNPs associated with the phenotype under consideration; each study has a corresponding Cohort, which includes the information about the groups of people from which the biological sample is collected, gathered to study a specific phenotypic trait (trait_name attribute). Specifically, the regions represented within the Item are obtained by comparing the DNA sequences of cases (people affected by the phenotype) with controls (people not showing that phenotype). An Item may represent a sample at different stages (initial or replication); each study may be based upon groups of individuals or on trios. The Cohort entity stores the cardinalities of the cases, controls, individuals or trios that provide the corresponding item, both of the initial stage or replicate stage(s). A Cohort can reference many Ancestries, each containing given ancestral information about the represented partition, e.g., the country of origin, the ancestral category or the country from which the participants are selected.

This module required an ad-hoc implementation to create the computational structure for the novel GWAS view of the model. Note that each GWAS study has one cohort, but can have more than one ancestry; to indicate this, we append an ordinal number to all the attributes referring to ancestries (e.g., broad_ancestral_category_[0_9]). As an example, let us consider the metadata file of the GCST007269 study; as its cohort is linked to seven different Ancestries, we will include the metadata pairs: $\langle$broad_ancestral_category_1, European$\rangle$, $\langle$broad_ancestral_category_2, Asian unspecified$\rangle$, ..., $\langle$broad_ancestral_category_7, European$\rangle$. This allows us to create, correspondingly, seven referenced rows in the Ancestry table.

### Semantic Enrichment

During this step, the trait_name of the Cohort table (extracted from the output of the Mapper) is associated with controlled terms, lists of synonyms and hyperonyms, and external links to reference ontologies. The result of this phase complements the information contained in the database of metadata. The adoption of a specific knowledge base for this attribute provides us with value normalization. Using external knowledge bases is essential in the biomedical domain, where specialized ontologies are already available and well-recognized. This process is supervised and requires a preliminary selection of the most suitable ontologies to describe the attribute (as previously applied to other attributes [19, 29]).

Ontological access to genomic data is currently well-supported by several search services, which are capable to integrate a high number of ontologies. As a broker search service to the underlying ontologies, we chose the Ontology Lookup Service (OLS [30]) by EMBL-EBI^2^. OLS provides ontology search, visualization, and ontology-based services. The accepted input is a keyword, the provided result is a list of annotations. In the API request, a *fieldList* parameter can be used to specify the specific elements to be included in the output along with other formatting preferences.

For each distinct value of the relational database trait_name field, resulting from the union of all the traits from GWAS Catalog and FinnGen, we perform one call to OLS API whose results is stored in the following form: the original value (called raw); possible parsed values deriving from a simple syntactic pre-processing of raw (e.g., removal of punctuation, split of long expressions...); the $\langle$ontology,ontology_id$\rangle$ pair, uniquely identifying an ontological term within a service; pref_label and synonym, respectively the primary textual expression used for the term and its alternative version; score, information regarding the goodness of a match: 10, when there is a perfect match with a pref_label, 9 with a synonym.

In total, from OLS, we were able to retrieve 4,694 original trait_name raw values to be enriched, which resulted into 5,145 distinct parsed values (a portion of original values were split by comma). Out of  120K API calls performed on OLS, about one half found partial/exact matches with terms in 232 different ontologies. Such matches were used for calculating more advanced scores. An excerpt of the results is shown in Table 1. We calculate the match_score as a measure of how well a term from the ontology matches a value: we subtract from the initial score (10 or 9) the distance between the raw value and the label retrieved from the services (either pref_label o synonym).

**Table 1:** We collect the results of each call to OLS API in a table that contains: raw value (input value to the program, before parsing—not shown here); parsed value (input value after parsing the raw value); ontology (used to annotate the input value—not shown here); ontology_id (id of the term in the ontology used for annotating the parsed value); pref_label (preferred label of the ontological term used for the annotation); synonym (a list of synonyms associated to the term corresponding to the ontology id); match_score (where (P) and (S) respectively indicate that the score was calculated subtracting a penalty from the 10 or 9 initial match scores); onto_suitability; onto_acceptance; annotation_score. The table shows an excerpt of our results, ordered by descending annotation score

parsed	ontology	pref		match	onto	onto	annot.
value	id	label	synonyms	score	suit.	acc.	score
creatinine meas.	NCIT_C64547	Creatinine Measurement	Creatinine, Creatinine Level, ...	10 (P)	4.39	0.86	5.23
creatinine meas.	NCIT_C61048	Urine Creatinine Measurement	Urine Creatinine Measurement	9 (P)	4.39	0.86	4.71
mean arterial pressure	NCIT_C120935	Mean Pulmonary Arterial Pressure	MPAP, Mean Pulmonary Arterial Pressure	9 (P)	4.39	0.86	4.71
diverticulitis	EFO_1001460	diverticulitis	digestive tract diverticulum inflammation, ...	10 (P)	3.40	0.32	3.36
survival time	EFO_0000714	survival time	survival, time of survival	10 (P)	3.40	0.32	3.36
diastolic blood pressure	EFO_0006336	diastolic blood pressure	DIABP, diastolic pressure	10 (P)	3.40	0.32	3.36
viral load	EFO_0010125	viral load	viral titer, viral titre, viral burden	10 (P)	3.40	0.32	3.36
mean corpuscular hemoglobin	EFO_0004527	mean corpuscular hemoglobin	MCH, mean corpuscular haemoglobin	10 (P)	3.40	0.32	3.36
calcium measurement	EFO_0004838	calcium measurement	calcium levels	10 (P)	3.40	0.32	3.36
autoimmune disease	EFO_0005140	autoimmune disease	autoimmunity	10 (P)	3.40	0.32	3.36
moderate albuminuria	HP_0012594	Moderate albuminuria	High urine albumin levels, Microalbuminuria	10 (P)	3.40	0.32	3.36
glomerular filtration rate	EFO_0005208	glomerular filtration rate	GFR	10 (P)	3.40	0.32	3.36
anxiety	EFO_0005230	anxiety		10 (P)	3.40	0.32	3.36
diaphragmatic hernia	EFO_0007216	congenital diaphragmatic hernia	CDH, congenital diaphragmatic hernia, ...	9 (P)	3.40	0.32	3.02
sarcoidosis	EFO_0010723	ocular sarcoidosis		9 (P)	3.40	0.32	3.02
sneeze	EFO_0007887	autosomal dominant compelling helio...	photic sneeze reflex, Peroutka sneeze	8 (S)	3.40	0.32	2.69
anorexia nervosa	HP_0002039	Anorexia	Anorexia	8 (P)	3.40	0.32	2.69
lean body mass	NCIT_C139219	Lean Body Mass to Total Body Mass Ratio	Lean Body Mass to Total Body Mass Ratio, ...	5 (P)	4.39	0.86	2.62
fasting blood insulin meas.	EFO_0004465	fasting blood glucose meas.	fasting glucose-related traits, ...	7.5 (P)	3.40	0.32	2.52
protozoal diseases	MONDO_0001955	protozoal dysentery		7.5 (P)	3.40	0.26	2.43
primary sclerosing	EFO_0004268	sclerosing cholangitis	fibrosing cholangitis, cholangitis, sclerosing, ...	7 (P)	3.40	0.32	2.35
event free survival time	EFO_0004919	metastasis free survival	metastasis free survival time	6.5 (S)	3.40	0.32	2.18
response to vancomycin	NCIT_C76312	Vancomycin Resistant Enterococcus	Vancomycin-Resistant Enterococcus, VRE, ...	4 (P)	4.39	0.86	2.09
fish oil supplement exposure meas.	EFO_0009116	vitamin supplement exposure measurement	vitamin use exposure measurement	5.5 (P)	3.40	0.32	1.85
magnesium:creatinine ratio meas.	EFO_0007635	concentration dose ratio	CDR measurement	4.5 (S)	3.40	0.32	1.51
other and unspecified	EFO_0009734	unspecified juvenile idiopathic arthritis	unspecified JIA, ...	4 (S)	3.40	0.32	1.34
pre-eclampsia	DOID_10591	pre-eclampsia	gestational hypertension, ...	10 (P)	0.14	0.39	0.89
bipolar disorder	DOID_3312	bipolar disorder	bipolar depression, manic disorder, ...	10 (P)	0.09	0.39	0.85
binocular movement	MP_0006148	binocular blindness		7.5 (P)	0.59	0.28	0.77
asthma	HP_0002099	Asthma	Bronchial asthma, Asthma	10 (P)	0.16	0.31	0.75
stroke	SYMP_0000734	stroke	cerebral accident, brain attack, apoplexy, ...	10 (P)	0.10	0.25	0.58
schizophrenia	OMIT_0013465	Schizophrenia, Paranoid		9 (P)	0.27	0.18	0.52
schizophrenia	OMIT_0013464	Schizophrenia, Disorganized		9 (P)	0.27	0.18	0.52
creatinine measurement	MAXO_0000832	serum creatinine measurement		9 (P)	0.31	0.13	0.46
bipolar disorder	NBO_0000258	bipolar disorder	BD, manic depression, bipolar affective disorder	10 (P)	0.08	0.19	0.44
postydysenteric arthropathy	MPATH_684	arthropathy		8 (P)	0.05	0.19	0.34
inflammatory biomarker meas.	MAXO_0000554	interleukin-1 beta biomarker measurement	IL-1 beta assessment	6.5 (P)	0.31	0.13	0.33

The distance is computed using the principle of the Needleman-Wunsch algorithm [34]; in the original algorithm, the input is represented by two strings whose letters need to be aligned. We adapted the algorihm to ‘align’ words rather than letters. The total distance is calculated as a sum of distances between words where a match contributes 0 distance; a swap (when two consecutive words trade places) 0.5 distance; an insertion 1 distance; a deletion 2 distance; and a mismatch 2.5 distance. The algorithm minimizes the number of deletions and prefers swaps to indels or mismatches (Table 1).

Each ontology is scored from two perspectives: i) the onto_acceptance, i.e., how well-known and trusted the ontology is by the biomedical community (retrieved through Recommender Web Services [32]^3^); ii) the onto_suitability, i.e., how much the ontology is adequate for annotating traits. For a given ontology, suitability is calculated as the product of: a) the coverage (percentage of raw values successfully annotated by the ontology); b) the sum of the match_scores associated to all obtained annotations, normalized by the number of total annotations. Intuitively, the score will be higher if the ontology annotates more terms with pref_labels rather than with synonyms.

Finally, for each annotation, i.e., the mapping between a parsed value and an ontology term (ontology_id), we compute an overall annotation_score by multiplying each raw value’s match_score by a linear combination of onto_suitability and onto_acceptance. Based on the annotation_scores obtained for each parsed value using different ontologies, we informed the service evaluation phase. Specifically, we aggregate results by grouping on specific ontologies, thereby computing the *Coverage* as the percentage of raw values that are found in each ontology; the *Score* as the average match_score of all the annotated attribute values weighted by the onto_acceptance; the *Suitability* as the measure of the adequacy of the ontology to annotate the attribute values. Since most of the times only one ontology does not provide an acceptable coverage for all the attribute values, we also compute a small set of ontologies to annotate values. Our algorithm first tries to match values only with the first (most appropriate) ontology, then tries to match only the values left unmatched with the following ontologies, until a fixed point is found for coverage. As a consequence, we compute the *SetCoverage*, *SetScore*, and *SetSuitability* metrics, corresponding to these small sets of ontologies.

### Flattening

Results of the transformation, mapping, and enrichment stages are fed back to the file-based representation of metadata (in GDM format), so that the pipelines that use this representation can exploit the understanding, modeling, and integration efforts that have been applied on GWAS information.

## Results

The Methods have presented all the steps to reach a complete integration of the datasets of two selected data sources within the META-BASE repository. Measurable results are produced at the end of the Semantic Enrichment and of the Flattening. In this section, we provide a preliminary measurement of how the proposed steps contributed to resolving two main needs: i) the lack of interoperability between phenotypic traits among GWAS sources and cross-data-type source; ii) the lack of solutions for processing GWAS studies with other genomic signals. To address the first point, we propose a systematization of the enrichment process, allowing GWAS phenotypic traits to be connected to arbitrary ontologies, either selected through an automatic evaluation process or specified by users; to address the second point, we enable the possibility to query GWAS datasets by means of the GenoMetric Query Language (GMQL [20]), overcoming heterogeneity between GWAS sources and other kinds of genomic sources.

### Semantic Enrichment

#### Ontology Selection

The results of our selection, based on the calculations presented in the Methods ‘Semantic Enrichment’ section, are shown in Table 2, where we indicate the preferred ontology sets with three indicators: *SetCoverage*, *SetScore*, and *SetSuitability*. Note that a second preferred ontology is added when the first one did not reach 0.85 coverage; in such case, indicators refer to the union of the ontologies. Additional file 4 contains the complete table with the scores associated to all the computed sets of one, two, and three ontologies.

**Table 2:** Results of ontology evaluation and selection process

	Preferred ontologies	SetCoverage	SetScore	SetSuitability
Best for coverage	EFO	0.801	1.430	3.405
Best for score/suit.	NCIT	0.777	2.947	4.387
Best pair for coverage	EFO, NCIT	0.928	1.638	3.540
Best pair for score/suit.	NCIT, ENM	0.875	2.624	3.919
Best triplet for coverage	EFO, NCIT, SNOMED	0.969	1.694	3.576

As a final outcome, we choose the Experimental Factor Ontology (EFO) and the National Cancer Institute Thesaurus (NCIT [36]) to annotate the trait values of our sources. Note that we sacrificed coverage (by not choosing the triplet of ontologies EFO, NCIT, SNOMED [37]) to prefer a minimal set of ontologies that already reaches acceptable results. Note that the choice of EFO and NCIT is also consistent with the history of the data sources (i.e., GWAS Catalog traits are originally curated with EFO), and also guarantees interoperability with the GCM disease field, which is also enriched using the NCIT.

#### Enrichment Process

After selecting the ontology set, we proceed with the enrichment of the trait values. The process is supported by an interactive tool^4^ that annotates values with concepts from the chosen ontologies and allows to handle expert user feedback when annotations have a low matching score: users can either accept one of the proposed solutions, or manually specify new annotations.

**Fig. 3:**
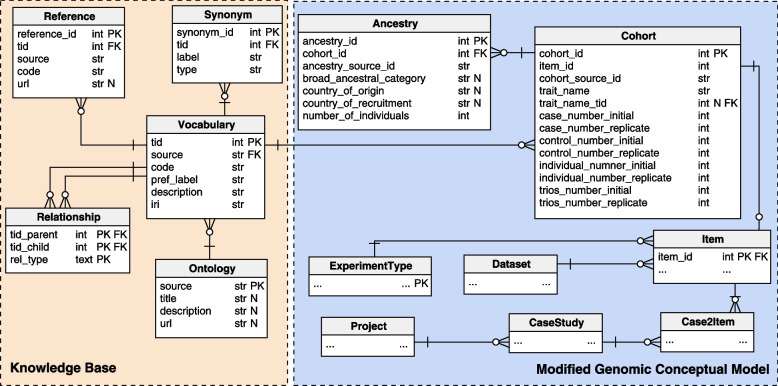
Logical schema of the database for handling GWAS datasets

The result of the enrichment is contained within the relational database described in the logical schema of Fig. 3, whose blue part represents the tables from the GCM (of which we only show in detail the ones which have ontological attributes), and whose orange part (called Knowledge Base) is populated from ontologies and referenced from the trait_name attribute; it stores all the information retrieved from OLS services and relevant to annotate our values. The main tables of the Knowledge Base are: Vocabulary, Synonym, containing alternative labels that can be used as synonyms of the preferred label, Reference, containing references to equivalent terms from other ontologies, Ontology, a table presenting details on the specialized ontologies; Relationship, containing ontological hierarchies between terms and the type of the relationships.

The GCM attribute trait_name is equipped with a companion-attribute trait_name_tid that references the ontological term in the vocabulary table. Value enrichment is a supervised procedure: for each value associated to a trait_name, the system initially looks for a suitable term in the Knowledge Base; if a match is available the procedure is completed. Else, a match is searched on the specified ontologies (EFO and NCIT) on OLS. Once the term has been selected, we populate the tables of the Knowledge Base with all the information derived from OLS regarding the term: description, iri, synonyms, xrefs, hyperonyms and hyponyms (both of is_a and part_of kinds). The depths of ancestors and descendants retrieved from the ontology are configurable by constant specification.

With the current implementation and data, the automatic enrichment process successfully annotates the 63% of original raw values, meaning that this fraction of the input values is annotated with ontological terms that reach a match_score of at least 5 (out of 10, i.e., perfect match with a preferred label). The remaining non-annotated values can be handled using a manual curation procedure, which supports the expert user by providing suggestions (e.g., terms for which a low match score was found). In any case, a manual annotation can always be provided. So far, we enriched attribute values by linking them to 3,004 terms, 1,877 from EFO and 1,127 from NCIT. In addition to terms that directly annotate values, we included all terms that could be reached by traversing up to three ontology levels from the base term.

#### Semantic overlap across data sources

By means of the semantic enrichment process, which took in input 3,276 distinct traits from the GWAS Catalog and 2,778 traits (endpoints) from FinnGen, we were able to find 90 common concepts (i.e., ontological terms that are referenced by – possibly several – distinct datasets both in GWAS Catalog and FinnGen); see their list in Additional file 5. Interestingly, the NCIT terms used for annotation allowed us also to make connection with diseases present in the metadata of other data sources present in the META-BASE repository. Namely, we had 4 matches with ENCODE [38] datasets, regarding ‘colorectal carcinoma’, ‘hepatocellular adenocarcinoma’, ‘hepatocellular carcinoma’, and ‘squamous cell carcinoma; mesothelioma’. Similarly, we had 5 matches with The Cancer Genome Atlas [39] datasets, regarding ‘Cholangiocarcinoma’, ‘Esophageal Carcinoma’, ‘Head and Neck Squamous Cell Carcinoma’, ‘Liver Hepatocellular Carcinoma’, ‘Lung Adenocarcinoma’, showing potential for our approach and indicating the possibility to use the new GWAS datasets together with processed data describing other genomic signals.

**Fig. 4:**
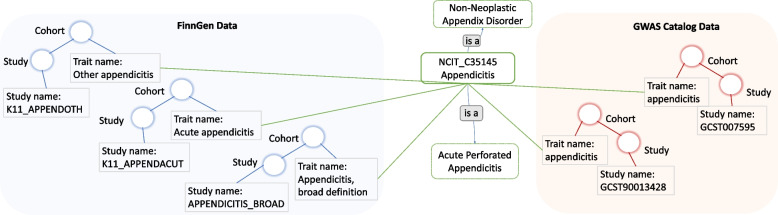
Enrichment of different Cohorts trait names’ values

Figure 4 shows the possibility to process together the SNPs from three FinnGen endpoints and two GWAS Catalog studies as they all refer to phenotype concepts that concern the general concept of ‘appendicitis’, captured by the C35145 term of the NCI Thesaurus.

### Datasets interoperability

Genome-wide association studies inform on the correlations between many phenotypes and their corresponding mutations of DNA. The exact interpretation of that SNPs is not trivial for two reasons: 1) the outputs of GWASs are often large clusters of SNPs in linkage disequilibrium, making it difficult to distinguish causal SNPs from neutral variants in linkage; 2) even assuming that the causal variants can be identified, interpretation is limited by incomplete knowledge of non-coding regulatory elements, their mechanisms of action and the cellular states and processes in which they function. For the aforementioned reasons, it becomes important to further investigate GWAS data by merging and analyzing different genomic datasets.

In this section, we show examples of application of the GenoMetric Query Language (GMQL [20]) on the GWAS standardized data, highlighting the advantages of our data representation in terms of information retrieval and integrative processing. GMQL is a closed algebra over datasets with the ability of computing distance-related queries along the genome, seen as a sequence of positions. GMQL is capable of expressing high-level queries for genomic computations and executes them on big datasets over a cloud computing system [21] (employing Apache Spark [40] as its backbone), specific for genomic data processing. The GMQL system [41] contains a multiplicity of public genomic datasets from a variety of sources, ready to be used within tertiary analysis pipelines; it features datasets from sources such as ENCODE, The Cancer Genome Atlas [39], Roadmap Epigenomics [42], and 1000 Genomes [43], among others. GWAS Catalog and FinnGen datasets (available at http://gmql.eu/gwas/) can be easily uploaded in the GMQL system private space of any user and processed together with the ones in the GMQL repository (as shown, e.g., in [44]).

In the following, we propose four use cases along with their GMQL queries (which can be alternatively expressed using the Python [45] or R [46] packages); we focus on query aspects, acting on both region data and metadata, which highlight the strengths of the datasets produced by our work. For further details about the reported GMQL operators, the interested readers can refer to [47].

#### Breast cancer GWAS SNPs on relevant genes

The Cancer Genome Atlas (TCGA [39]) gathers multiple genomic datasets related to 37 different types of cancer; these include gene expression profiling, copy number variation profiling, SNP genotyping, genome wide DNA methylation profiling, microRNA profiling, and exon sequencing. TCGA has been converted to GDM-compliant format in OpenGDC [48], then imported within the GMQL repository. Mapping SNPs indentified by GWAS onto TCGA profiles of gene expression for a given type of cancer can support a better understanding of given cancer types’ risk factors. For breast cancer data, we map highly expressed genes from TCGA dataset onto SNPs from GWAS, we focus on the genes BRCA1 and BRCA2, as germline mutations in those genes are the main part of genetic and hereditary factors for breast cancer [49]; we finally extract only regions having at least one overlapping SNP taken from GWAS studies mapped to the same trait.

**Code snippet 1:**
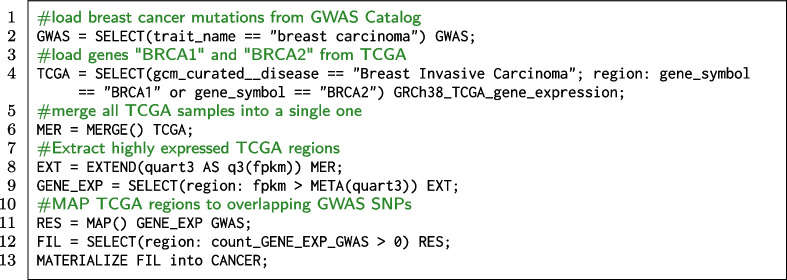
GMQL query extracting highly expressed regions of BRCA1 and BRCA2 genes harbouring GWAS SNPs associated to breast carcinoma

The GMQL query in the Code snippet 1 loads the studies from GWAS Catalog mapped to the trait ‘breast carcinoma’ (line 2) and the data referred to genes BRCA1 and BRCA2 from the GRCh38_TCGA_gene_expression dataset (line 4), which are merged within a single sample grouping all the regions from the TCGA samples (line 6). Then, the metadata of such sample are extended with an additional attribute that represents the third quartile of FPKM^5^ (i.e., the value above which only 25% regions fall), line 8. Such value is used as a threshold to extract only regions that are highly expressed (line 9). Line 11 presents the core operation of the query: MAP compares the regions of the GENE_EXP dataset (called *reference*) with the GWAS dataset of SNPs (called *experiment*). The result reports all the regions of the dataset GENE_EXP, equipped with counts of how many SNPs they overlap with (when the count is positive, see line 12).

A typical row of the result materialized by line 13 has the following form: $\langle$ chr, left, right, gene, fpkm, count_snps, quart3 $\rangle$ = $\langle$ chr13, 32315473, 32400266, BRCA2, 347792385, 1, 283041330.3 $\rangle$. This example query takes about 7 minutes and returns 5 samples with a total of 440 regions (17.24 MB). All the regions in the output are referred to the gene BRCA2; no overlapping SNPs are found for gene BRCA1. The resulting regions can be further processed using bioinformatics pipelines or computational approaches that combine them with the results of other studies, for instance allowing to prioritize positions of interest for a more complete explanation of breast cancer mechanisms (e.g., distant metastasis [50], cancer predisposition [51], or promoter activity [52]).

#### GWAS SNPs occurring on untranslated regions

The GENCODE consortium [53] provides manual annotations of the human genome and it is the reference for annotations adopted by most large international consortia including ENCODE and TCGA. Among other annotations (comprising protein-coding genes, pseudogenes, long non-coding RNAs, and small non-coding RNAs), we focus on untranslated regions (UTRs). Genetic variants in the coding sequence of a gene (exons) have often been given priority (because of their easier interpretation). Nevertheless, it has been known for long that coding sequence variants *per se* are insufficient for mapping complex diseases. Variants in the intervening sequences (introns) or in the untranslated regions (UTRs) – although not changing the predicted protein sequence – may instead be pivotal in the regulation of gene expression [54]. The UTRs are the mRNA sequences flanking the beginning and end of the coding sequences; as their name suggests, UTRs are part of the mRNA but are not translated into proteins. Mutations occurring in UTRs are difficult to interpret and associated with consequences.

**Code snippet 2:**
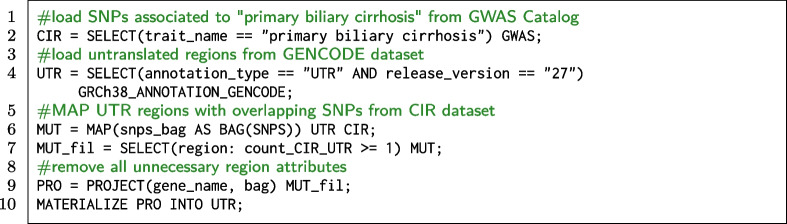
GMQL query that extracts UTR regions on which SNPs associated to the “primary biliary cirhosis” trait occur.

**Fig. 5:**
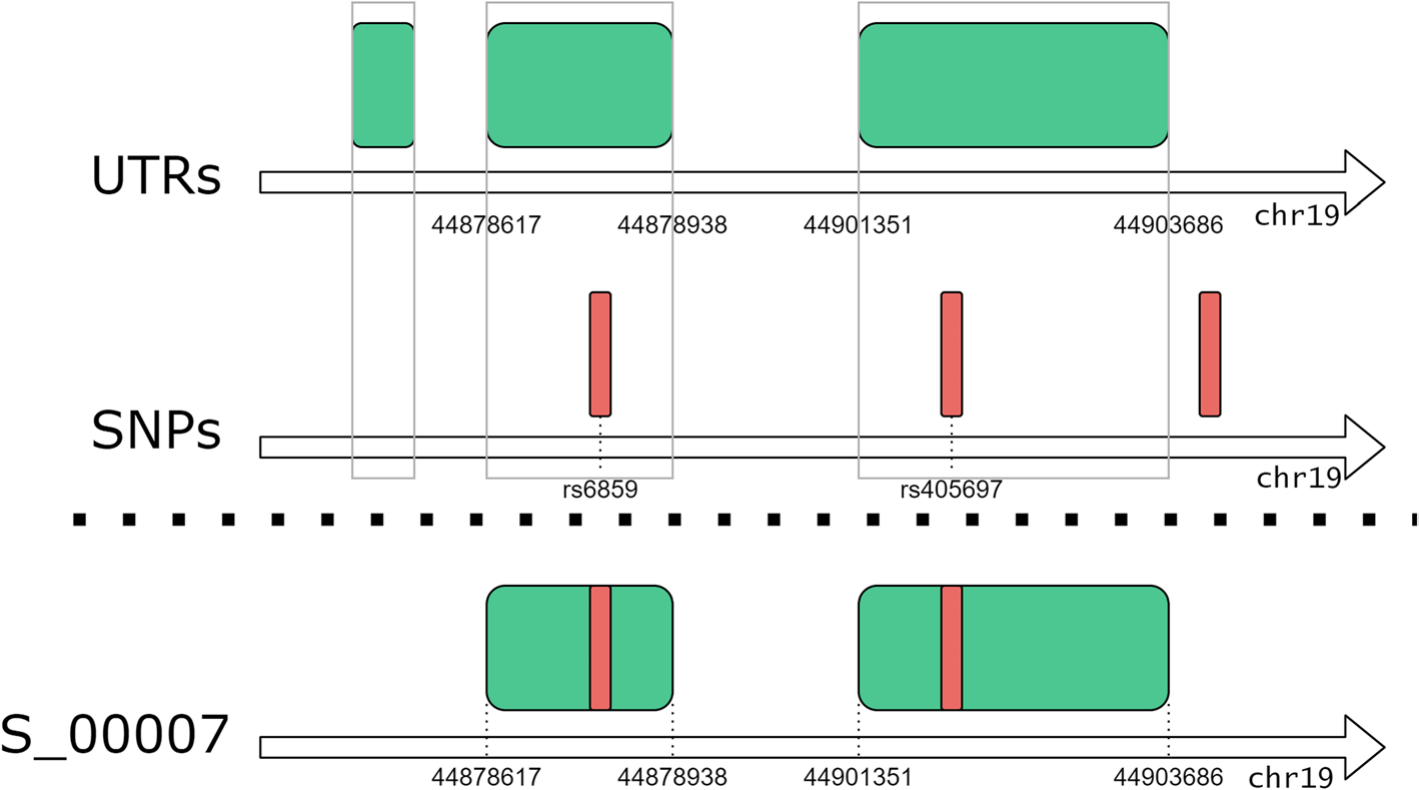
Visual representation of the GMQL query for SNPs occurring in untranslated regions. Green rectangles represent UTRs from GENCODE dataset, while the red stripes are the SNPs mapped to a GWAS trait. The query extracts only those UTRs that have at least one overlapping SNP

With GMQL we can contribute to explore this issue by allowing, for arbitrary GWAS traits, to quickly map all SNPs onto UTRs, as they are annotated in GENCODE. The GMQL query reported in the Code snippet 2 selects GWAS Catalog studies mapped to the “primary biliary cirrhosis” trait (line 2) and the UTR regions from the latest release of the GENCODE dataset (line 4). The MAP operation (line 6) extracts, for each region in the UTR dataset, the overlapping SNPs (which are listed in a new region attribute called snps_bag); Line 7 statement selects in the output dataset only the SNPs that occur in UTR regions. Finally line 9 extracts the UTRs only projecting their useful attributes (i.e., gene_name and the just calculated snps_bag). Figure 5 captures visually the operations performed by the query. For “primary biliary cirrhosis” we materialized (line 10) 21 UTR regions (distributed over 3 samples) with significant SNPs. The query can be iterated on different traits, e.g., “coronary artery disease” (135 UTRs, 18 samples), “Alzheimer’s disease” (36 UTRs, 10 samples), or “bipolar disorder” (30 UTRs, 9 samples). Query processing takes times that vary from a few minutes to over an hour, depending on the size of the samples.

Note that the MAP operation at line 6 could have been used for the dual purpose of extracting SNPs falling within UTRs, instead of extracting UTRs hosting at least one SNP. This can be easily achieved by using the statement MAP () CIR UTR, where the two datasets are swapped. When executed in this way, the query extracts SNPs that deserve further analysis. Indeed, genetic variation happening in UTRs could impact regulatory elements that affect the interaction of the UTRs with proteins or microRNAs. Among the consequences on functions, there are the change of mRNA transcription, translation, and access to regulators. Alterations of these regulatory mechanisms are known to modify molecular pathways and cellular processes, potentially leading to disease processes [54, 55]. It is thus very important to allow for a systematic identification of such SNPs, which can then be linked to the affected functionalities, e.g., as done in [56] for the specific case of polyadenylation signals. In our demonstrative example result set, we found the *rs2189521* mutation, occurring in gene *IL21R*: Qiu et al. [57] reported that the risk allele for primary biliary cirrhosis regulates differential IL21R expression; this variant is also highly correlated with multiple SNPs in the IL21R region, suggesting that variation in IL21R expression may explain this signal. By applying several histochemical experiments, they showed that the enhanced expression in PBC livers (in the hepatic portal tracks) of IL21R and of its ligand, IL21, support an involvement of IL21 signalling pathway deregulation in the disease mechanism.

#### Match GWAS mutations with variants from 1000 Genomes Project

Genome-wide association studies can discover new loci that contribute to common human diseases. For each locus, it is currently necessary to sequence the newly discovered region to define all common and rare variants. GWASs carried on so far explained a modest fraction of all the disease risks; part of these unexplained risks are due to alleles with lower frequencies but probably larger effects. If such alleles are in genes that were already localized by GWAS, then targeted sequencing may find them. Similarly, some of the unexplained risks are due to the effects of structural variants that are not in linkage disequilibrium with common SNPs. Thus, a more complete understanding of the role of genetic variation in disease requires a deeper catalog of genetic variation.

GWAS data can be usefully compared with referenced data, e.g., 1000 Genomes [43], a project born in 2008 as an international research effort to establish the most detailed catalogue of human genetic variations by far [58]. The genomes sequenced in the 1000 Genomes Projects are not categorized with regard to phenotype, but provide a resource of variants to support deeper understanding of newly discovered loci influencing human disease. The META-BASE repository contains the full 1000 Genomes biallelic SNP and indel variants aligned to the reference genome GRCh38. The projects include SNPs with allele frequencies as low as 1% across the genome and 0.1-0.5% in gene regions, as well as structural variants like CNVs. It includes genomes from 26 different populations, comprising the Finnish one. We thus formulate our GMQL query as the one that finds, for each relevant SNP from the FinnGen study associated to Schizophrenia, the closest deletion from the 1000 Genomes dataset referred to Finnish people.

**Code snippet 3:**
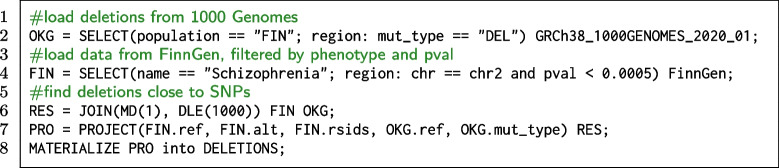
GMQL query that extracts deletions typical of the Finnish population that are close to significant Schizophrenia SNPs

**Fig. 6:**
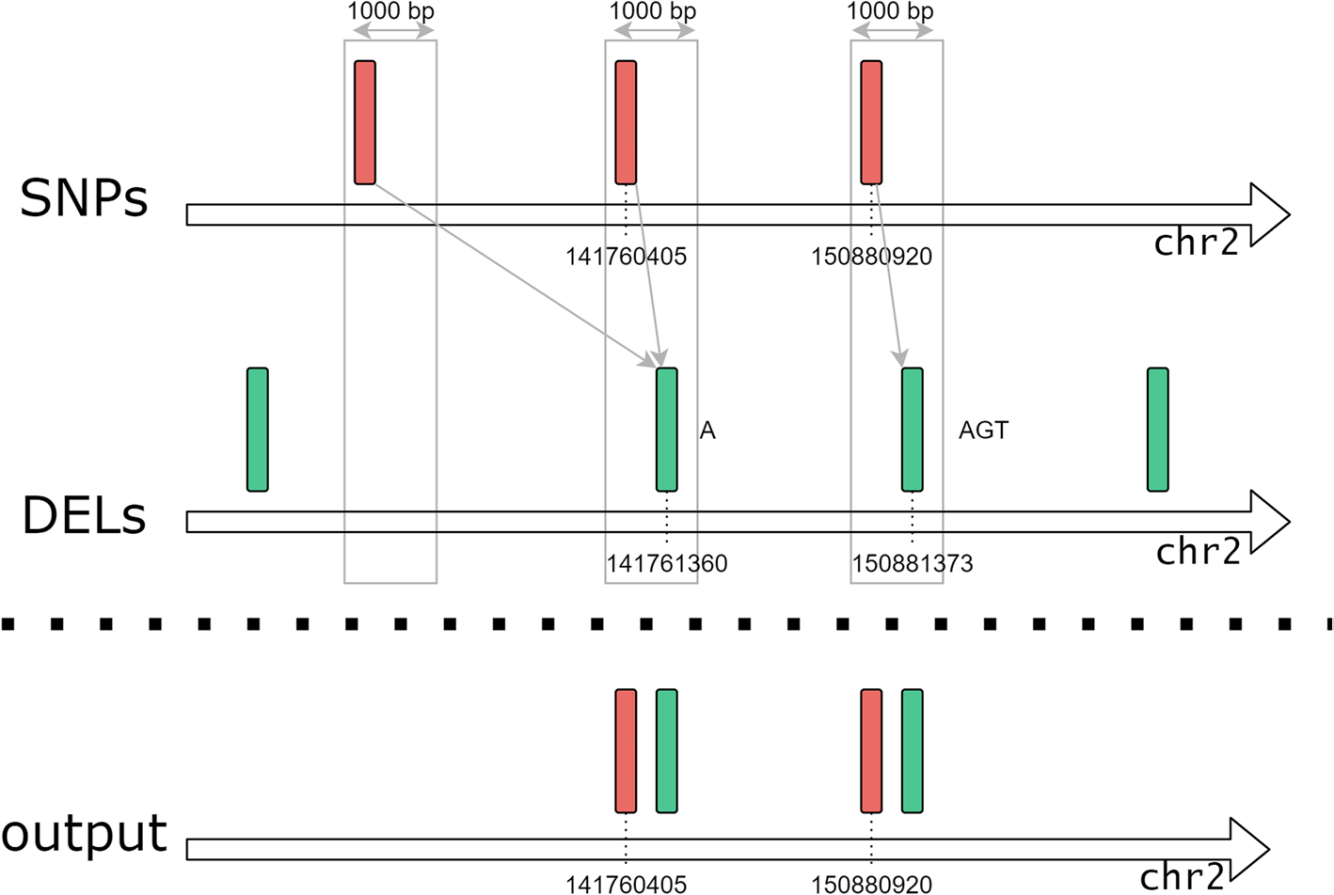
Visual representation of the GMQL query that evaluates the distance between regions of two samples, applying a genometric condition. The green regions are the deletions identified from 1000 Genomes Projects while the red ones are the SNPs taken from FinnGen dataset. For each deletion, the query considers the closest SNP, which is kept only when falling within 1000 base pairs from the considered deletion

The GMQL code is reported in the Code snippet 3: line 2 selects the samples from 1000 Genomes dataset referred to the Finnish population. For those samples, it filters only regions that represent deletions. Line 4 selects the samples from FinnGen dataset referring to Schizophrenia phenotype. From the resulting sample, it filters the regions based on a reasonably low *p*-value. Line 6 uses the JOIN operator to find, for each pair of samples – one from the FIN dataset and one from the OKG dataset – the closest deletion from each FinnGen SNP only if its distance is less than 1000 base pairs from the SNP. Line 7 exploits the operator PROJECT to remove superfluous region attributes, keeping only the relevant ones. The query process is visually represented in Fig. 6.

A typical row of the result materialized by line 8 has the following form: $\langle$ chr, left, right, F.ref, F.alt, F.rsids, O.ref, O.m_type $\rangle$ = $\langle$ chr2, 150880920, 150881373, G, A, rs149379995, AGT, DEL $\rangle$. The attributes F.ref, F.alt and F.rsids derive from FinnGen dataset while O.ref and O.m_type are from 1000 Genomes.

Note that if a user alternatively requested instead variations that are overlapping relevant SNPs, a different JOIN condition may be used: JOIN(distance<1; output: BOTH). In this way we would find, for each variation from 1000 Genomes dataset, the overlapping SNPs from FIN dataset. Then, only variation of SNP type should be selected: SELECT(region: OKG.mut_type == “SNP”) RES. Note that similar queries may be iterated on other populations/cohorts, such as the japanese one (1000 Genomes: population “JPT”; GWAS Catalog: country_of_recruitment “Japan”); the chinese one (1000 Genomes: population == “CHB”, “CHS”, and “CDX”; GWAS Catalog: country_of_recruitment “China”); or the United Kingdom one (1000 Genomes population: “GBR”, “ITU”, and “STU”; GWAS Catalog country_of_recruitment: “U.K.”), allowing to process thousands of regions at the same time. This query demonstrates the possibility to systematically compare locations of GWAS and reference panels of variation in healthy populations. GWAS-derived SNPs have – to date – been used to impute about 2.5 million SNPs in the HapMap Project (HapMap) [59]. However, it has been observed that low-frequency and rare variants are not well covered in the HapMap panel, whereas recently released versions of the 1000 Genomes Project are more comprehensive [60]. Our repository includes both GWAS information and 1000 Genomes Project in the same format, thereby allowing position-based reduction of the space of search, possibly to be exploited for later imputation tasks.

#### Mutations occurring in cell-specific enhancers

In [61] the authors developed a new fine-mapping algorithm to identify candidate causal variants for 21 autoimmune diseases from genotyping data. They found out that about 60% of likely causal variants map to enhancer-like elements, with preferential correspondence to stimulus dependent CD41 T-cell enhancers that respond to immune activation by increasing histone acetylation and transcribing non-coding RNAs. Unfortunately, it is not trivial to associate the enhancer with its corresponding gene, since it is situated within some hundreds of kilobases from the gene that it regulates. The study can be extended to many different human cell lines, attempting to verify whether mutations that occur in cell specific enhancers are related with any particular disease or trait.

**Fig. 7:**
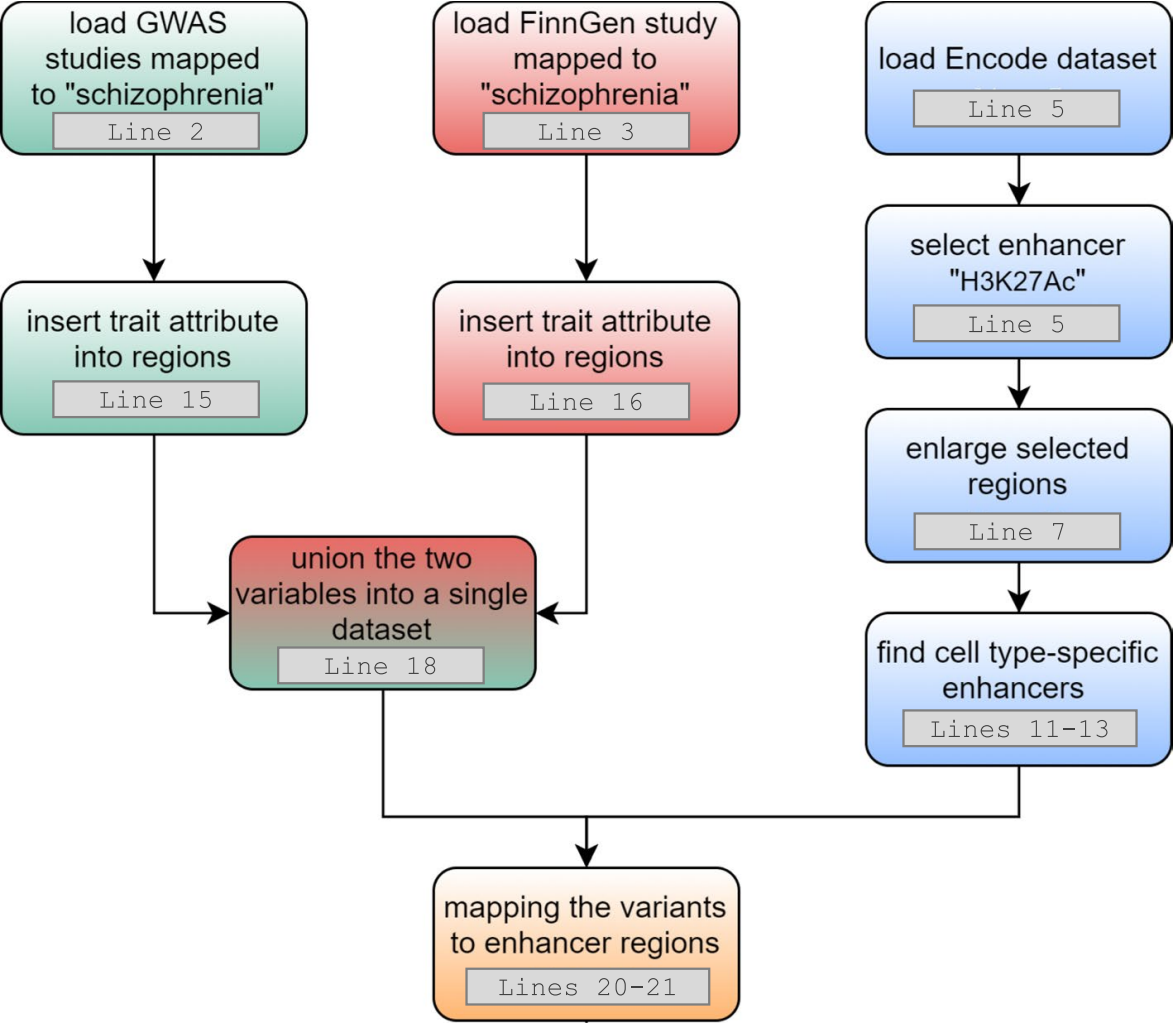
Execution flow of the proposed GMQL query. The three datasets are first pre-processed separately. The studies from GWAS Catalog and FinnGen are unified into a single dataset and then enhancer regions from ENCODE dataset are mapped into regions from the unified one

The computational experiment can be formulated as a GMQL query that exploits GWAS mutations and enhancer regions from ENCODE. Pinoli’s experiment [62] focuses on a particular histone modification, i.e., the acetylation at the 27th lysine residue of the histone protein 3 (H3K27Ac), which can be captured by a ChIP-seq experiment. The modification H3K27Ac is defined as active enhancer mark since it is known to encourage enhancer activation. The query workflow outlined in Fig. 7 aims to find the mutations occurring in cell-specific enhancers and to understand the resulting disease or phenotypic trait. We employ both datasets integrated in this work; for demonstration purposes, we here focus only on schizofrenia-related traits.

**Code snippet 4:**
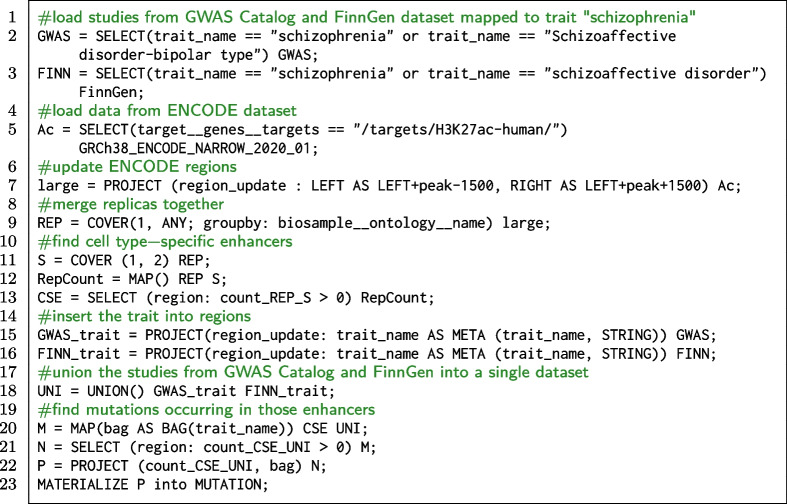
GMQL query extracting mutations occurring in cell-specific enhancers

The GMQL code is reported in Code snippet 4: lines 2–3 hold the instructions to upload the studies mapped to schizophrenia from GWAS Catalog and FinnGen datasets. Line 5 loads the GRCh38_ENCODE_NARROW dataset, selecting DNA regions that are enriched by H3K27Ac. Line 7 allows to update the coordinates of the previously selected ENCODE regions, enlarging them by 3000 base pairs around the peaks; this operation defines the enhancer regions. Line 9 applies the operator COVER over the ENCODE samples, using the groupby option. It computes the result grouping the input dataset samples by the values of their biosample__ontology__name metadata attribute. Lines 11, 12 and 13 filter the regions that are cell type-specific enhancers. To distinguish cell type-specific enhancers from shared ones, we considered their frequency; we are looking for those peaks of H3K27Ac that occur in no more than two cell lines among all the samples that we considered. The COVER (1, 2) operation considers all areas defined by a minimum of one overlapping region up to two of them; output region attributes include only region coordinates. The operation MAP () allows to retrieve the original regions with all their region attributes, adding the information of their frequency. Finally, using the SELECT operator we extract only the regions identified in line 12. Lines 15 and 16 exploit the operator PROJECT to add the new region attribute trait_name in each region (copied from the homonymous metadata attribute). Line 18 creates a dataset called UNI containing all the samples from GWAS_trait and FINN_trait datasets. Lines 20–21 contain the core operations of the query. The MAP operator adds to each region of the ENCODE dataset a counter corresponding to the number of overlapping regions of UNI dataset. The option bag adds a further region attribute with a list of values of the attribute trait_name of the mapped GWAS regions. The operator SELECT extracts only those ENCODE regions that have at least one corresponding GWAS mutation. Line 22 allows to keep in the output regions only the coordinates and two relevant columns. As last operation, the dataset P is materialized so it can be downloaded and explored. Approximately, this complex query takes about 8h running time and outputs 39008 regions distributed over 318 samples (about 4GB of memory).

The GMQL query has two main purposes: i) filtering out only the truly causal variants (alternatively performed with fine-mapping algorithms); ii) identifying the variants that occur in non-coding regions, in particular the enhancer regions where the H3K27Ac modification occurred. It can be repeated on all the traits in GWAS Catalog and FinnGen, allowing to study whether mutations that occur in cell-specific enhancers are related with any particular disease or trait [62]. The list of traits from GWAS Catalog and FinnGen mapped onto common terms on EFO or NCIT ontologies (Additional file 5) may be used for performing a cross-source application of this study.

## Discussion

As genomic data continues its exponential growth [63], data management techniques must continuously be adapted to correctly handle the growing amounts and related heterogeneity. Many works exploit the conceptual modeling to capture the diverse biological objects and to interpret their relationships (see [64–68]). However, such works only contribute to the conceptual clarification of genomic entities, while they do not provide practical frameworks to extract novel knowledge from data. The Genomic Conceptual Model [18] goes further the entity description and proposes feasible data organization for complex biological integrated repositories; it poses the bases for an architecture that drives the integration of new genomic repositories [19]. The work presented in this article exemplifies how the architecture can be exploited to integrate new datasets, mapping them to a shared conceptual model. Here – expanding on [28] – we have presented the Extended Genomic Conceptual Model, holding a novel GWAS view ready to accommodate datasets that represent Genome-Wide Association Studies. GWAS are of great importance, being the widely-accepted means to discover genetic risk factors for common disease and other phenotypic traits. Towards a broader use of GWAS for genomic tertiary analysis, we have shown three main outputs, discussed next.

*Data integration.* We designed an integration process to include GWAS within META-BASE. This strategy can be re-applied with small effort on all GWAS datasets, even when organized in a structure different from the NHGRI-EBI GWAS Catalog and FinnGen. With reference to our pipeline (Fig. 1) it must be noted that while Downloader and Transformer are source-specific (requiring the implementation of ad-hoc modules for each incoming source), Mapper and Enricher only require small configuration changes in order to be reapplied to new sources; finally, Flattener is completely automatized. Integration workflows for genomic datasets have been previously proposed (a broad review has been conducted in [69]); a number of genomic actors have built integration efforts [70–73], but – to the best of our knowledge – this work is the first that expresses GWAS in the same format as diverse datasets such as the ones used for cancer genomics or epigenomics. The proposed solution work has only been possible thanks to the exploitation of a previous solid stream of research on data modeling and integration through a systematic approach [18, 19]. At the moment, the main bottleneck of our approach remains the time required to run the integration pipelines. Both FinnGen and GWAS Catalog regularly output updated datasets, thus requiring to rerun our workflow to obtain newly GDM-compliant datasets.

*Semantic Enrichment.* We proposed a method to semantically annotate traits with an automatic process. Semantic enrichment of metadata with appropriate ontologies [74] has been tackled both with source-independent methods [75, 76] and with source-specific ones [77, 78]. Several GWAS sources are already working in the direction of homogenizing their values and linking them to existing ontologies. However, we claim that until a shared standard is imposed, differences will not be overcome. Thus, it is important to expose methods that allow to automatically annotate (and re-annotate) traits following the indication of a set of ontologies that are deemed appropriate by the user. In this way, even traits coming from different sources can converge to same ontological representations. We do not modify original values; in fact, we make explicit their relationship with existing ontological terms. This choice allows to exploit a semantic search at different levels: by original values (returning only results from one source) or by common values (returning all the results from different sources that are mapped into the same terms). This strategy has been successfully applied in the GenoSurf [79] semantic search engine.

*Cross-data type processing.* Analysing together different signals of the genome (tertiary data analysis) is very powerful but is still mostly performed through ad hoc scripts (e.g., with BEDTools [80] or BEDOPS [81]). More sophisticated systems have been proposed (GROK [82], GORpipe [83], STQL [84]), but none of these allows to directly analyse the genomic sites identified by GWAS in the context of other genomic signals is important and paves the way to larger multi-omic studies. Our example queries in the context of the GMQL system, a cloud-based multi-sample processor, go in this direction, showing interesting biological findings. Note that the shown examples are of small scale to allow reproduction in short times: queries run on multiple traits and chromosomes may require long computational times. Note that we chose an orthogonal set of examples to show the possibility of using different GWAS datasets together or using (possibly multiple) GWAS datasets with other signals (TCGA, 1000 Genomes, ENCODE, and annotation data).

## Conclusion

GWASs bring important insights and outputs to the current genomic research. Their identified genomic regions have, however, rarely been analyzed in the context of other genomic information, including other mutations, epigenomic regions, or gene expressions. Being able to analyze GWAS data from multiple sources together with other processed genomic datasets is of high importance. We thus proposed to express GWAS datasets in the GDM format, compliant with the other sources included in the META-BASE repository. A purely model-driven integration effort achieves the inclusion GWAS datasets within a repository of other tertiary analysis processed datasets. We download, transform and map GWAS information within the integrated repository, also enriching the traits descriptions by means of several information from existing specialized biomedical ontologies, shortening the distance of different GWAS datasets; this process can be generalized to all kinds of genomic experiment descriptions that benefit from bio-ontology mapping [29, 85]. We demonstrated the proposed approach on two important GWAS data sources, organized according to heterogeneous data models, namely the NHGRI-EBI GWAS Catalog and FinnGen, while several other datasets can be added in the future with minimal effort.

For fully exploiting the achieved integration result, we make GWAS datasets usable together with other processed datasets (e.g., representing somatic and reference mutation data, genomic annotations, epigenetic signals) embedding them within a multi-sample processing system called GMQL [41]. This system allows to pursue important genomic tertiary data analysis tasks, able to respond to biological questions regarding positional properties of GWAS identified mutations.

## Supplementary information

**Additional file 1.** GWAS Catalog source files descriptions.

**Additional file 2.** FinnGen source files descriptions.

**Additional file 3.** Region data representation for GWAS Catalog and FinnGen.

**Additional file 4.** Results of ontology selection for trait_name annotation.

**Additional file 5.** Common traits and mapping to ontologies.

## Data Availability

The code of the integration framework, extended by the GWAS specific modules, is available at https://github.com/DEIB-GECO/Metadata-Manager; the employed Enricher framework is available at https://github.com/DEIB-GECO/Metadata-Enricher. All the files transformed in GDM format from GWAS Catalog and FinnGen endpoints are available at http://gmql.eu/gwas/gwas_catalog and http://gmql.eu/gwas/finngen. The GMQL queries can be run at http://genomic.deib.polimi.it/gmql-rest/.

## Footnotes

1 The META-BASE Scala [23] implementation is publicly available on the GitHub repository https://github.com/DEIB-GECO/Metadata-Manager.

2 Details on this choice are provided in [29]; considered alternatives are BioPortal [31], Recommender [32], and Zooma [33].

3 The Recommender Web Services calculate it from the number of visits to the ontology page in BioPortal and the presence or absence of the ontology in UMLS [35].

4 The GitHub repository of the Enricher tool is https://github.com/DEIB-GECO/Metadata-Enricher.

5 FPKM stands for Fragments Per Kilobase of transcript per Million mapped reads values of its regions.
