# Correction: Processing genome-wide association studies within a repository of heterogeneous genomic datasets

**DOI:** 10.1186/s12863-023-01135-4

**Published:** 2023-06-05

**Authors:** Anna Bernasconi, Arif Canakoglu, Federico Comolli

**Affiliations:** Department of Electronics, Information and Bioengineering, Politecnico di Milano, Via Ponzio 34/5, Milano, 20133 Italy

BMC Genomic Data (2023) 24:13

10.1186/s12863-023-01111-y

Following publication of the original article [1], it was reported that the article entitled “Processing genome-wide association studies within a repository of heterogeneous genomic datasets” was published in the regular issue of this journal instead of in the supplement issue.

The details of the supplement in which this article ought to have been published are given below:

About this supplement

This article has been published as part of BMC Genomic Data Volume 24 Supplement 1, 2023: Selected articles on Conceptual Modeling for Life Sciences (CMLS 2021 workshop and ER 2021 conference): genomic data. The full contents of the supplement are available online at https://bmcgenomdata.biomedcentral.com/articles/supplements/volume-24-supplement-1.

The publisher apologizes for any inconvenience caused.

