# Supplementary material for: Processing genome-wide association studies within a repository of heterogeneous genomic datasets
Source: BMC Genom Data. 2023 Mar 3;24:13. doi: 10.1186/s12863-023-01111-y (PMC9985298; doi:10.1186/s12863-023-01111-y)
Supplement: Supplementary file 1 — Additional file 1. GWAS Catalog source files descriptions. [file 12863_2023_1111_MOESM1_ESM.pdf]

Additional File 1

Anna Bernasconi [1], Arif Canakoglu [1], and Federico Comolli[1]

[1]Dept. of Electronics, Information and Bioengineering (DEIB), Politecnico di Milano, 20133 Milano, Italy

GWAS Catalog source files descriptions

Below is the list of attributes provided in the three files 'Associations.tsv'[AS], 'Studies.tsv' [S], and 'Ancestry' [AN]. For each attribute, we indicate the symbols of the files in which it appears and a brief description.

- DATE ADDED TO CATALOG [AS][S]: the date in which a study is published in the Catalog.
- PUBMEDID [AS][S][AN]: PubMed identification number.
- FIRST AUTHOR [AS][S][AN]: last name and initials of first author.
- DATE [AS][S][AN]: publication date of the study online.
- JOURNAL [AS][S]: abbreviated journal name in which the study is published.
- LINK [AS][S]: PubMed URL of the study.
- STUDY [AS][S]: title of paper.
- DISEASE/TRAIT [AS][S]: disease or trait examined in study.
- INITIAL SAMPLE DESCRIPTION [AS][S][AN]: sample size and ancestry description for initial stage of the study.
- REPLICATION SAMPLE DESCRIPTION [AS][S][AN]: sample size and ancestry description for subsequent replication(s) of the study.
- REGION [AS]: cytogenetic region associated with the SNP.
- CHR_ID [AS]: chromosome number associated with the SNP.
- CHR_POS [AS]: position in the chromosome of the SNP.
- REPORTED GENE(S) [AS]: gene(s) reported by author.
- MAPPED GENE(S) [AS]: gene(s) mapped to the strongest SNP. If the SNP is located within a gene, that gene is reported. If the SNP is located within multiple genes, these genes are listed separated by commas. If the SNP is intergenic, the upstream and downstream genes are listed, separated by a hyphen.
- UPSTREAM_GENE_ID [AS]: entrez Gene ID for nearest upstream gene to rs number, if not within gene.

- DOWNSTREAM_GENE_ID [AS]: entrez Gene ID for nearest downstream gene to rs number, if not within gene.
- SNP_GENE_IDS [AS]: entrez Gene ID, if rs number within gene; multiple genes denote overlapping transcripts.
- UPSTREAM_GENE_DISTANCE [AS]: distance in kb for nearest upstream gene to rs number, if not within gene.
- DOWNSTREAM_GENE_DISTANCE [AS]: distance in kb for nearest downstream gene to rs number, if not within gene.
- STRONGEST_SNP_RISK_ALLELE [AS]: SNP(s) most strongly associated with trait + risk allele (? for unknown risk allele). May also refer to a haplotype.
- SNPS [AS]: strongest SNP; if a haplotype it may include more than one rs number.
- MERGED [AS]: denotes whether the SNP has been merged into a subsequent rs record (0 = no, 1 = yes).
- SNP_ID_CURRENT [AS]: current rs number (will differ from strongest SNP when merged = 1).
- CONTEXT [AS]: SNP functional class.
- INTERGENIC [AS]: denotes whether SNP is in intergenic region (0 = no, 1 = yes).
- RISK ALLELE FREQUENCY [AS]: reported risk allele frequency associated with strongest SNP in controls.
- P-VALUE [AS]: reported p-value for strongest SNP risk allele.
- PVALUE_MLOG [AS]: -log(p-value).
- P-VALUE (TEXT) [AS]: information describing context of p-value.
- OR or BETA [AS]: reported odds ratio or beta-coefficient associated with strongest SNP risk allele.
- 95% CI (TEXT) [AS]: reported 95% confidence interval associated with strongest SNP risk allele, along with unit in the case of beta-coefficients.
- PLATFORM (SNPS PASSING QC) [AS][S]: genotyping platform manufacturer used in initial stage.
- CNV [AS]: study of copy number variation (yes/no).
- ASSOCIATION COUNT [S]: number of associations identified for this study.
- MAPPED_TRAIT [AS][S]: trait mapped over the Experimental Factor Ontology.
- MAPPED_TRAIT_URI [AS][S]: URI of the EFO trait.
- STUDY ACCESSION [AS][S][AN]: accession ID allocated to a GWAS Catalog study.
- GENOTYPING_TECHNOLOGY [AS][S]: genotyping technology used in this study, with additional array information in brackets.
- STAGE [A]: stage of the GWAS to which the sample description is referred (initial, replication).
- NUMBER OF INDIVIDUALS [AN]: number of individuals in this sample.

- BROAD ANCESTRAL CATEGORY [AN]: broad ancestral category to which the individuals in the sample belong.
- COUNTRY OF ORIGIN [AN]: country of origin of the individuals in the sample.
- COUNTRY OF RECRUITMENT [AN]: country of recruitment of the individuals in the sample.
- ADDITIONAL ANCESTRY DESCRIPTION [AN]: any additional ancestry descriptors relevant to the sample description.

Table 1: Excerpt of the information reported in the 'Studies.tsv' file for study accession GCST005097.

PUBMEDID	FIRST AUTHOR	JOURNAL	STUDY	DISEASE-TRAIT	ASS. COUNT
29170203	Alonso N	Ann Rheum Dis	Identification of a novel locus on chromosome 2q13...	Fractures (vertebral)	3

Table 2: Excerpt of the information reported in the 'Ancestry.tsv' file for study accession GCST005097.

STUDY ACCESSION	INITIAL SAMPLE DESCRIPTION	REPLICATION SAMPLE DESCRIPTION	STAGE	NUMBER OF INDIVIDUALS	COUNTRY OF RECRUITMENT
GCST005097	1,553 cases; 4,340 controls	1,028 cases; 3,762 controls	replication	2799	U.K., Italy, Spain
GCST005097	1,553 cases; 4,340 controls	1,028 cases; 3,762 controls	replication	1991	U.K.
GCST005097	1,553 cases; 4,340 controls	1,028 cases; 3,762 controls	initial	5893	Australia, Denmark, U.K., Slovenia, Spain

Table 3: Excerpt of the information reported in the 'Associations.tsv' file for study accession GCST005097.

REGION	CHR_ID	CHR_POS	MAPPED GENE(S)	SNPS	P-VALUE
15q26.1	15	92464744	ST8SIA2	rs2290492	3*10 -7
2q13	2	112192944	AC092645.1 - ZC3H8	rs10190845	1*10 -9
11q12.1	11	57980425	OR5BD1P - CYCSP26	rs7121756	4*10 -7
