# Supplementary material for: Processing genome-wide association studies within a repository of heterogeneous genomic datasets
Source: BMC Genom Data. 2023 Mar 3;24:13. doi: 10.1186/s12863-023-01111-y (PMC9985298; doi:10.1186/s12863-023-01111-y)
Supplement: Supplementary file 2 — Additional file 2. FinnGen source files descriptions. [file 12863_2023_1111_MOESM2_ESM.pdf]

Additional File 2

Anna Bernasconi [1], Arif Canakoglu [1], and Federico Comolli[1]

[1]Dept. of Electronics, Information and Bioengineering (DEIB), Politecnico di Milano, 20133 Milano, Italy

FinnGen source files descriptions Below is the list of attributes of the FinnGen summary statistics with a brief explanation of their meaning. For each of the, we specify if the information is provided in the Manifest file [M] or directly in the specific endpoint file [E].

- phenocode [M]: alphanumeric code given to a phenotype.
- name [M]: complete name of the phenotype.
- n_cases [M]: cardinality of the cases group for the associations of the current trait.
- n_controls [M]: cardinality of the controls group for the associations of the current trait.
- path_bucket [M]: path of the current file for Google cloud-based access.
- path_https [M]: path of the current file for command-line access.
- #chrom [E]: chromosome on build GRCh38.
- pos [E]: position in base pairs on build GRCh38.
- ref [E]: reference allele.
- alt [E]: alternative allele (effect allele).
- rsids [E]: variant identifier.
- nearest_genes [E]: nearest gene name from variant.
- pval [E]: p-value from SAIGE.
- beta [E]: effect size estimated with SAIGE for the alternative allele.
- sebeta [E]: standard deviation of effect size estimated with SAIGE.
- maf [E]: alternative (effect) allele frequency.
- maf_cases [E]: alternative (effect) allele frequency among cases.
- maf_controls [E]: alternative (effect) allele frequency among controls.

Table 1: Excerpt of Manifest file of FinnGen repository Release 5.

phenocode	name	n_cases	n_controls	path_bucket	path_https
F5_SCHIZO	Schizophrenia, schizotypal and delusional disorders	7999	168900	gs:// ... F5_SCHI-ZO.gz	https:// ... F5_SCHI-ZO.gz
G6_PARKIN-SON	Parkinson's disease	1587	175312	gs:// ... G6_PAR-KINSON.gz	https:// ... G6_PAR-KINSON.gz
TUBERCU-LOSIS	Tuberculosis	801	176098	gs:// ... TUBERCULOSIS.gz	https:// ... TUBERCULOSIS.gz

Table 2: Excerpt of the TUBERCULOSIS.gz file (FinnGen provides one file for each trait).

#chrom	pos	ref	alt	rsid	pval
1	115637	G	A	rs74337086	0.8316
1	216439193	T	C	rs571377638	0.7225

Table 3: Excerpt of the F5_SCHIZO.gz file (FinnGen provides one file for each trait).

#chrom	pos	ref	alt	rsid	pval
1	133855	C	T	rs528106901	0.5142
1	195798153	A	C	rs2942912	0.655
