# Supplementary material for: Processing genome-wide association studies within a repository of heterogeneous genomic datasets
Source: BMC Genom Data. 2023 Mar 3;24:13. doi: 10.1186/s12863-023-01111-y (PMC9985298; doi:10.1186/s12863-023-01111-y)
Supplement: Supplementary file 3 — Additional file 3. Region data representation for GWAS Catalog and FinnGen. [file 12863_2023_1111_MOESM3_ESM.pdf]

Additional File 3

Anna Bernasconi[1], Arif Canakoglu[1], and Federico Comolli[1]

[1]Dept. of Electronics, Information and Bioengineering (DEIB), Politecnico di Milano, 20133 Milano, Italy

Region data representation for GWAS Catalog and FinnGen

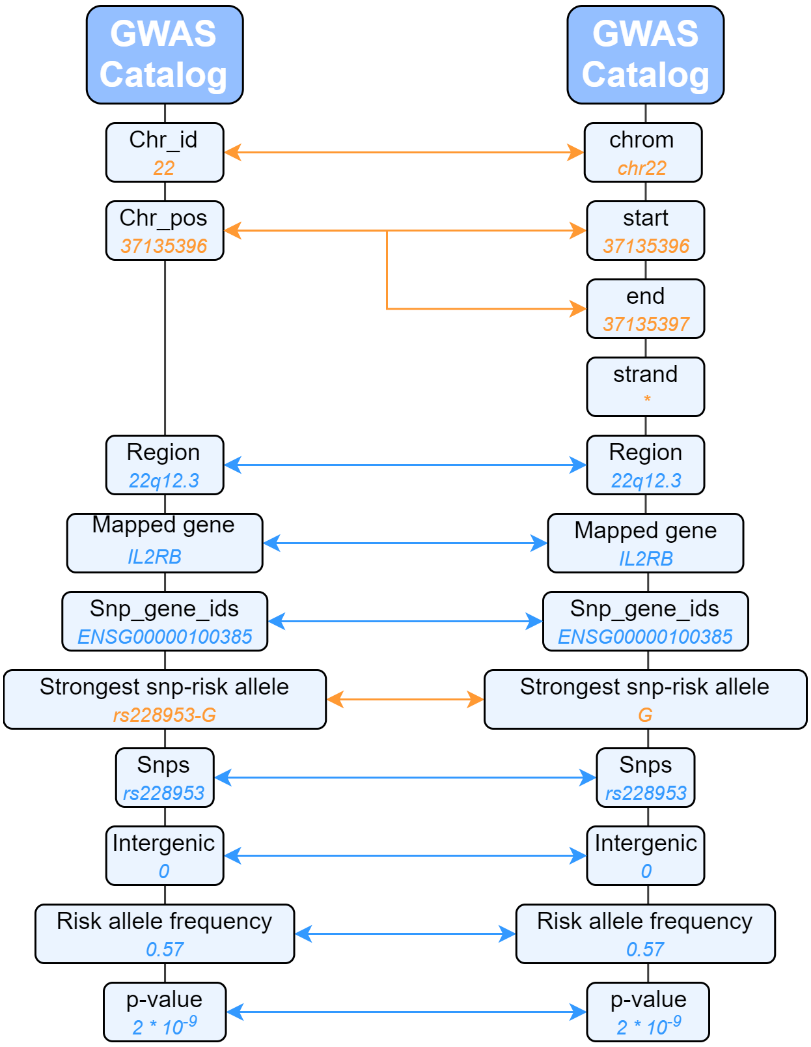

Figure 1: Transformation of region data of GWAS Catalog into the Genomic Data Model format. Each attribute is coupled with an example value; the color *orange* represents values that are modified, while the *light blue* represents unchanged values. Please note that in this diagram are reported only some relevant region attributes of GWAS Catalog; for the full list refer to Additional File 1.

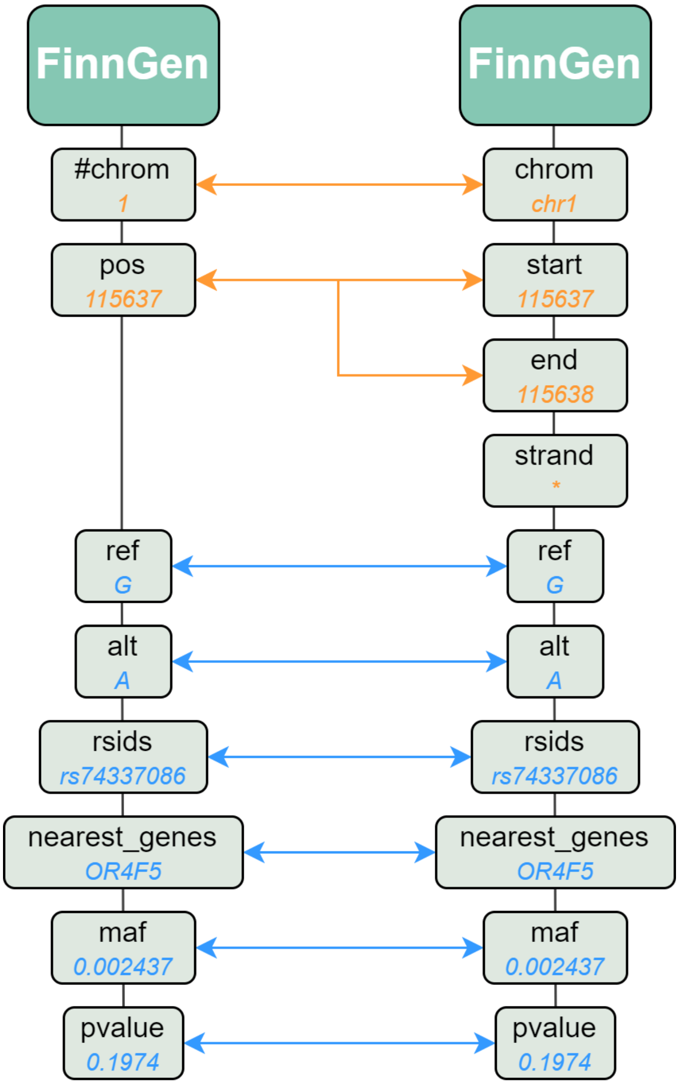

Figure 2: Transformation of region data of FinnGen into the Genomic Data Model format. The notation is the same of Figure 1. In this figure are reported only some relevant region attributes of FinnGen, for the full list, refer to Additional File 2.

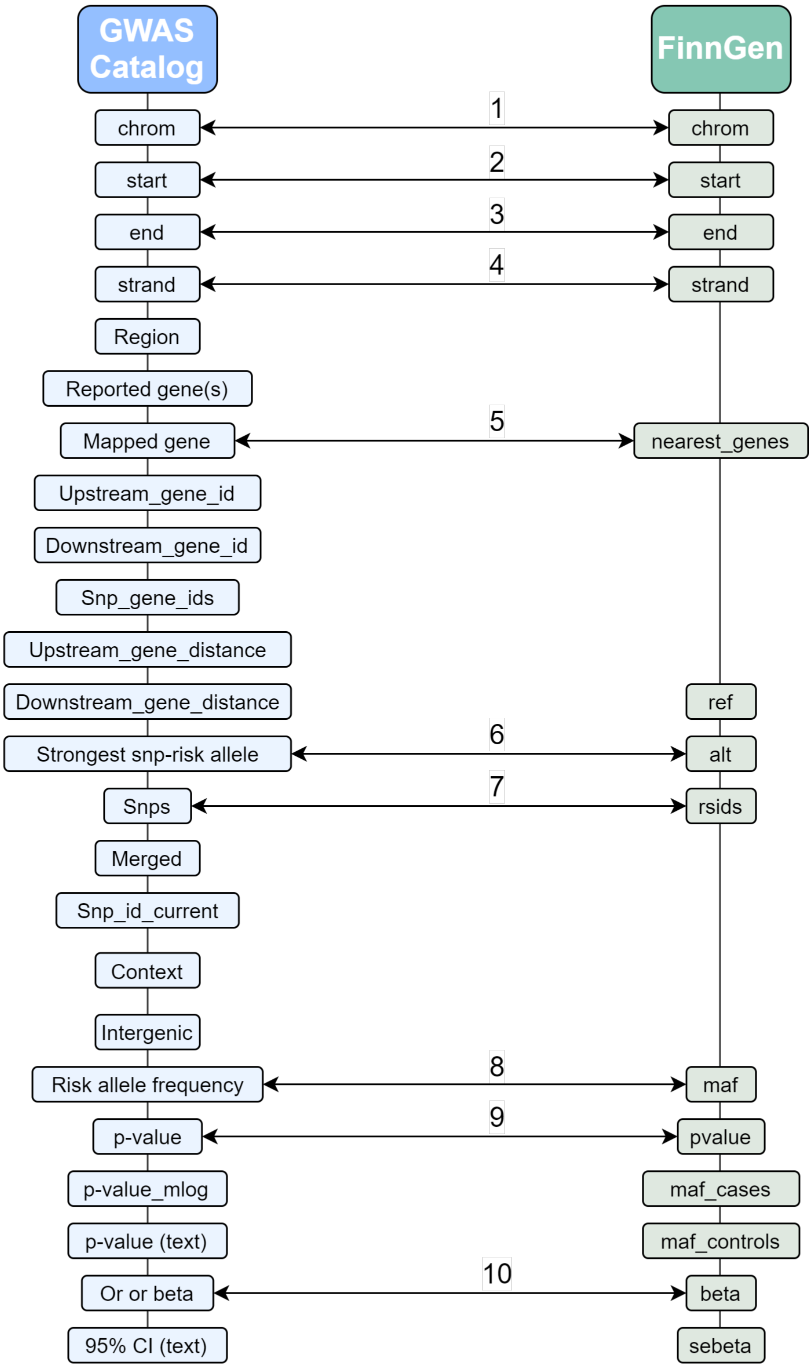

Figure 3: Correspondences between the region attributes of two considered GWAS data sources.
