# Supplementary material for: Processing genome-wide association studies within a repository of heterogeneous genomic datasets
Source: BMC Genom Data. 2023 Mar 3;24:13. doi: 10.1186/s12863-023-01111-y (PMC9985298; doi:10.1186/s12863-023-01111-y)
Supplement: Supplementary file 4 — Additional file 4. Results of ontology selection for trait_name annotation. [file 12863_2023_1111_MOESM4_ESM.pdf]

Additional File 4

Anna Bernasconi [1], Arif Canakoglu [1], and Federico Comolli [1]

[1]Dept. of Electronics, Information and Bioengineering (DEIB), Politecnico di Milano, 20133 Milano, Italy

Results of Ontology Selection for TraitName Annotation

**Ontologies set**	**SetCoverage**	**SetScore**	**SetSuitability**	#Ontologies
efo, ncit, snomed	0.9688898359	1.693520724	3.575633002	3
ncit, snomed, enm	0.9544001705	2.408982017	3.673432407	3
efo, ncit, hp	0.9424675048	1.658377469	3.552878289	3
efo, ncit, ordo	0.9411890049	1.656626941	3.551744849	3
efo, snomed, omit	0.939910505	1.223317816	2.978080944	3
efo, ncit, enm	0.9396974217	1.654578638	3.550418605	3
efo, ncit, omit	0.9377796719	1.651935531	3.548707234	3
efo, ncit, mondo	0.9375665885	1.651641185	3.54851665	3
efo, snomed, hp	0.9373535052	1.226555658	2.984770549	3
efo, ncit, doid	0.9367142553	1.650462462	3.547753445	3
efo, snomed, ordo	0.9367142553	1.22736788	2.986448657	3
efo, ncit, bao	0.9347965054	1.647802475	3.546031145	3
efo, snomed, mondo	0.931813339	1.233631942	2.999390644	3
efo, snomed, enm	0.9303217558	1.235551495	3.003356575	3
efo, snomed, doid	0.9288301726	1.237477214	3.007335243	3
efo, snomed, bao	0.9281909227	1.238304416	3.009044301	3
efo, ncit	0.927764756	1.63795511	3.539655128	2
ncit, mondo, enm	0.9224376731	2.491682389	3.738613443	3
efo, snomed	0.9211591732	1.247479419	3.028000493	2
ncit, snomed, hp	0.9164713403	2.507173907	3.803700154	3
ncit, hp, enm	0.9160451737	2.590872809	3.917784858	3
ncit, snomed, mondo	0.9105050075	2.523364465	3.825179601	3
ncit, snomed, ordo	0.9096526742	2.525694739	3.828271095	3
ncit, snomed, omit	0.9085872576	2.52861373	3.832143618	3
ncit, enm, doid	0.9070956744	2.532458254	3.787515465	3
ncit, enm, omit	0.9036863414	2.54188192	3.801147821	3
ncit, snomed, bao	0.9021947582	2.546272455	3.85557083	3
ncit, snomed, doid	0.9013424249	2.548645876	3.858719566	3
ncit, enm, ordo	0.8985723418	2.556151501	3.821790318	3
ncit, enm, bao	0.8983592585	2.556749592	3.822655523	3
ncit, snomed	0.8930321756	2.572024164	3.889734723	2

snomed, mondo, enm	0.8834434264	0.03735402004	0.438396565	3
efo, hp, omit	0.8817387599	1.352049223	3.208624503	3
efo, hp, ordo	0.8789686768	1.354469636	3.214726123	3
efo, mondo, hp	0.8753462604	1.31113176	3.135017276	3
ncit, enm	0.8751331771	2.623687637	3.919488672	2
ncit, mondo, hp	0.8738546772	2.628014114	3.932812898	3
efo, mondo, omit	0.8704453441	1.318291469	3.151285842	3
efo, omit, ordo	0.8698060942	1.319621321	3.155058489	3
efo, hp, enm	0.8695930109	1.362776226	3.235666205	3
ncit, hp, omit	0.8687406776	2.700148832	4.06182356	3
efo, hp, doid	0.8678883443	1.364305797	3.2395221	3
ncit, mondo, omit	0.864265928	2.656732779	3.973721563	3
ncit, hp, ordo	0.8634135947	2.713204826	4.079032901	3
efo, hp, bao	0.8634135947	1.368349657	3.249716266	3
ncit, hp, doid	0.8617089282	2.717416837	4.084584829	3
efo, mondo, ordo	0.8612827616	1.331895579	3.182197622	3
efo, omit, doid	0.8608565949	1.332878531	3.185091789	3
efo, enm, omit	0.8608565949	1.332191613	3.178643593	3
efo, mondo, enm	0.8589388451	1.33542232	3.190211219	3
snomed, hp, enm	0.8589388451	0.1964229573	0.74400984	3
ncit, hp, bao	0.8587257618	2.724828094	4.094353742	3
efo, omit, bao	0.8551033454	1.34154756	3.20473088	3
ncit, mondo, ordo	0.8536117622	2.689399124	4.020253553	3
efo, hp	0.8536117622	1.377355785	3.272419812	2
snomed, enm, omit	0.8533986789	0.0358667115	0.4246216574	3
efo, doid, ordo	0.8525463456	1.346677311	3.209503053	3
efo	0.8005540166	1.429934244	3.404964841	1
ncit	0.7779671852	2.947057945	4.387278926	1
snomed	0.6079266993	0.03637910278	0.5257830812	1
mondo	0.4466226294	0.03950517159	0.2455785212	1
hp	0.3573407202	0.5840334132	1.272533561	1
enm	0.3528659706	0.03459774247	0.1740890688	1
omit	0.3481781377	0.04440392584	0.2661410612	1
doid	0.2908587258	0.06472527922	0.19987215	1
bao	0.2130833156	0.5602390943	0.8819518432	1
ordo	0.1792030684	0.07838470685	0.4190283401	1
mp	0.1753675687	0.3458608113	0.5923716173	1
cmo	0.1564031536	0.00007678547271	0.01225229065	1
uo	0.1331770722	0.000001336458555	0.001491583209	1
oae	0.1233752397	0.006041673797	0.09652674196	1
mmo	0.1174089069	0.00004950029836	0.008523332623	1
scdo	0.1097379075	0.1934290217	0.3218623482	1
maxo	0.09972299169	0.1554180793	0.3083315576	1
chiro	0.09119965907	0.1486627981	0.2461112295	1
co	0.08779032602	0.01697385488	0.1364798636	1
gsso	0.08502024291	0.06070210109	0.08992115917	1
cido	0.0807585766	0.1415502537	0.1639676113	1
ecto	0.07393991051	0.04951962915	0.2139356488	1

uberon	0.06903899425	0.0003119434514	0.0164074153	1
to	0.06307266141	0.00206807436	0.0403792883	1
fma	0.06072874494	0.02156853245	0.127956531	1
rexo	0.05966332836	0.00216323704	0.04016620499	1
reto	0.05923716173	0.001473187527	0.03302791391	1
eupath	0.0588109951	0.001691711677	0.03526528873	1
symp	0.05859791178	0.1023585597	0.1020669082	1
atol	0.05774557852	0.0003778100854	0.01651395696	1
cco	0.056040912	0.004116089087	0.05369699553	1
chebi	0.05518857873	0.02097333826	0.1202855316	1
clo	0.05305774558	0.04096420525	0.03494566375	1
ontoneo	0.05241849563	0.1542239468	0.1436181547	1
bto	0.051992329	0.0005913236666	0.01960366503	1
go	0.05113999574	0.00008951274949	0.007564457703	1
mfomd	0.05071382911	0.07601370681	0.09226507564	1
cvdo	0.0492222459	0.000009759400341	0.002450458129	1
oba	0.04815682932	0.02063166946	0.111442574	1
om	0.04496057959	0.00001472395612	0.00287662476	1
ogg	0.04432132964	0.00007132144438	0.00628595781	1
pr	0.04346899638	0.0302329502	0.1281696143	1
mpath	0.04112507991	0.04653187252	0.04591945451	1
pw	0.04048582996	0.003464263685	0.04187087151	1
nbo	0.03750266354	0.09424390679	0.07841466013	1
pdro	0.03728958023	0	0	1
txpo	0.03494566375	0.07042390795	0.05156616237	1
dicom	0.03473258044	4.18E-07	0.0004261666312	1
htn	0.03451949712	0.06160250018	0.07852120179	1
dron	0.03324099723	0.005655586334	0.04847645429	1
gexo	0.03217558065	0.001981957064	0.02823353931	1
wbphenotype	0.03132324739	0.0003016216075	0.01086724909	1
genepio	0.03047091413	0.01694283556	0.08033240997	1
zp	0.0300447475	0.004884413636	0.04282974643	1
ohpi	0.02983166418	0.0677043956	0.06072874494	1
covoc	0.02940549755	0.02512542578	0.09610057532	1
obi	0.0287662476	0.0002122625502	0.008736415939	1
stato	0.0287662476	0.00001972993663	0.002663541445	1
hcao	0.02834008097	0.01165097914	0.06424461965	1
gecko	0.02791391434	0.001676331323	0.02418495632	1
hom	0.02706158108	0	0	1
ogsf	0.02642233113	1.37E-07	0.0002130833156	1
hpath	0.02578308118	0.0327301608	0.1027061581	1
planp	0.02556999787	0.009346153846	0.05465587045	1
ma	0.02407841466	0.0003923750115	0.01086724909	1
disdriv	0.02386533134	0	0	1
mfmo	0.02365224803	0.000003839339019	0.001065416578	1
opmi	0.02343916471	0.06186148616	0.04528020456	1
ogms	0.02279991477	0.00001756443779	0.002237374814	1
idomal	0.02258683145	0.001806946516	0.02258683145	1

micro	0.0219475815	0.006260967067	0.04144470488	1
zfa	0.0219475815	0.004981970669	0.03696995525	1
one	0.02088216493	0.01199852842	0.006818666098	1
mamo	0.02066908161	0.002068841288	0.02311953974	1
xao	0.0204559983	0.001775694296	0.02130833156	1
omp	0.02024291498	0.00006141285453	0.003942041338	1
rbo	0.01981674835	0.1007034041	0.05327082889	1
unimod	0.01981674835	0	0	1
ons	0.01960366503	0.02331421822	0.01150649904	1
sio	0.01939058172	0.1376819626	0.04687832943	1
eco	0.0191774984	0.00005800601368	0.003728958023	1
oostt	0.0191774984	1.89E-07	0.0002130833156	1
envo	0.01875133177	0.001534393584	0.01896441509	1
cl	0.01875133177	0.00006993006993	0.004048582996	1
gaz	0.01853824846	0.002613870989	0.02461112295	1
emapa	0.01832516514	0.0004955425944	0.01065416578	1
ncbitaxon	0.01789899851	0.001607459945	0.01896441509	1
fovt	0.01789899851	0.001571540186	0.01875133177	1
flopo	0.01747283188	0.04118278339	0.01683358193	1
pato	0.01704666525	0.09507718996	0.028020456	1
miro	0.01662049861	2.19E-07	0.0002130833156	1
xpo	0.01555508204	0.05319023319	0.03505220541	1
vt	0.01512891541	0.00473951315	0.02993820584	1
foodon	0.01427658214	0.001947963146	0.01864479011	1
nmrcv	0.01427658214	0.0001288040937	0.0047943746	1
omrse	0.01406349883	0.000003163964383	0.0007457916045	1
cio	0.01385041551	0	0	1
teddy	0.0136373322	0.0003170280205	0.007351374387	1
afo	0.01342424888	0.0128591384	0.0464521628	1
xco	0.01321116557	0.0006467422311	0.01033454081	1
ohd	0.01321116557	0.0001045482977	0.004155124654	1
bcio	0.01321116557	0.00001986486394	0.001811208182	1
edam	0.01299808225	0.2103517006	0.03878116343	1
aism	0.01299808225	0.00004024130812	0.002556999787	1
mf	0.01278499893	0.000113644435	0.004261666312	1
ido	0.01278499893	0.00006825768876	0.003302791391	1
cro	0.01257191562	2.89E-07	0.0002130833156	1
oarcs	0.0123588323	0	0	1
ovae	0.01193266567	0.0008876442117	0.01150649904	1
fypo	0.01171958236	0.007786374291	0.03377370552	1
ero	0.01150649904	0.0002564102564	0.006072874494	1
ogi	0.01108033241	0.0001003950237	0.003728958023	1
idocovid19	0.01086724909	0.000008356208454	0.001065416578	1
cdao	0.01086724909	0.000003008235043	0.0006392499467	1
reproduceme	0.01044108246	0.002817918133	0.0191774984	1
fideo	0.01044108246	3.48E-07	0.0002130833156	1
vo	0.01001491583	0.04121000676	0.008949499254	1
obcs	0.01001491583	0.00004388609563	0.002343916471	1

ohmi	0.009801832517	0.1009467384	0.03196249734	1
obib	0.009375665885	0.00004687832943	0.002343916471	1
geno	0.00916258257	0.00004370685682	0.002237374814	1
vido	0.008736415939	0.000001663089292	0.0004261666312	1
ms	0.008523332623	0.06781541658	0.01065416578	1
duo	0.008523332623	0	0	1
exo	0.008310249307	0.00001846722068	0.001385041551	1
ehdaa2	0.008097165992	0.0003032512028	0.005540166205	1
psdo	0.008097165992	0.00001357004273	0.001171958236	1
ecocore	0.007884082676	0.003097885868	0.01747283188	1
mfoem	0.007884082676	0.03668552934	0.005966332836	1
sibo	0.007884082676	0.007673155534	0.00287662476	1
clao	0.007670999361	0.0003712384876	0.005966332836	1
probonto	0.007670999361	0.00001704666525	0.001278499893	1
mco	0.00724483273	0.001516651834	0.01171958236	1
peco	0.007031749414	0.003183206452	0.01672704027	1
swo	0.006818666098	0	0	1
srao	0.006605582783	0.000008798278837	0.0008523332623	1
rxno	0.006392499467	0	0	1
opl	0.006179416152	0.00001469540107	0.001065416578	1
spd	0.005966332836	0.0001864479011	0.003728958023	1
apo	0.005966332836	0.0001109555265	0.00287662476	1
apollo	0.005966332836	0.00008766856412	0.002556999787	1
ontoavida	0.005966332836	0.000002435237892	0.0004261666312	1
omo	0.005966332836	6.09E-07	0.0002130833156	1
fbcv	0.005753249521	0.0007514738263	0.007351374387	1
pride	0.005753249521	0.00003093654063	0.001491583209	1
fbbt	0.005540166205	0.004792899408	0.01821862348	1
phipo	0.005540166205	0.0001285056303	0.002983166418	1
hao	0.005327082889	0.02130563818	0.003515874707	1
eol	0.005327082889	0.000002727466439	0.0004261666312	1
hso	0.005113999574	0.000008700902053	0.0007457916045	1
pco	0.004900916258	0.0001252559316	0.002770083102	1
iceo	0.004900916258	0	0	1
dideo	0.004900916258	0	0	1
nomen	0.004687832943	0.000003099393681	0.0004261666312	1
rnao	0.004687832943	7.75E-07	0.0002130833156	1
agro	0.004474749627	0.00003977555224	0.001491583209	1
sbo	0.004261666312	0.00009396974217	0.002237374814	1
tgma	0.004048582996	0.0001085603418	0.002343916471	1
sdgio	0.004048582996	0.00008971929077	0.002130833156	1
mi	0.004048582996	0	0	1
ceph	0.00383549968	0.0003979922816	0.004368207969	1
bco	0.00383549968	0.0001856192438	0.002983166418	1
cto	0.00383549968	0.00004640481095	0.001491583209	1
ro	0.00383549968	0	0	1
cdno	0.003622416365	0.004913450571	0.01491583209	1
ncro	0.003622416365	0.003065090686	0.0005327082889	1

rex	0.003409333049	0.0001534199872	0.002556999787	1
miapa	0.003409333049	0.00007697634775	0.001811208182	1
ino	0.003409333049	0.000009588749201	0.0006392499467	1
fobi	0.003409333049	0.000001065416578	0.0002130833156	1
iao	0.003196249734	0.00004091199659	0.001278499893	1
ecao	0.003196249734	0.00002301299808	0.0009588749201	1
plana	0.002983166418	0.001170131807	0.006605582783	1
dpo	0.002983166418	0.0007610118413	0.005327082889	1
omiabis	0.002983166418	0.0002739642629	0.003196249734	1
zeco	0.002983166418	0.00001095857052	0.0006392499467	1
cryoem	0.002983166418	0.00001095857052	0.0006392499467	1
ornaseq	0.002983166418	0.000001217618946	0.0002130833156	1
chmo	0.002770083102	0.09644883542	0.00628595781	1
aro	0.002770083102	0.04750278811	0.006179416152	1
upa	0.002770083102	0.00002098051107	0.0008523332623	1
rs	0.002556999787	0.0001026351303	0.001811208182	1
sepio	0.002343916471	0.000001549696841	0.0002130833156	1
fbbi	0.002343916471	0.000001549696841	0.0002130833156	1
lepao	0.002130833156	0.001152354571	0.005540166205	1
ico	0.002130833156	0.02596316216	0.001811208182	1
pso	0.002130833156	0.00008352865971	0.001491583209	1
po	0.002130833156	0.00004261666312	0.001065416578	1
mpio	0.002130833156	0.000001704666525	0.0002130833156	1
so	0.00191774984	0.0002504912754	0.002450458129	1
mod	0.00191774984	0.0000383549968	0.0009588749201	1
vto	0.001704666525	0.0001044108246	0.001491583209	1
fix	0.001704666525	0.000002130833156	0.0002130833156	1
taxrank	0.001491583209	0.00006088094731	0.001065416578	1
phi	0.001491583209	0.000002435237892	0.0002130833156	1
bfo	0.001491583209	0.000002435237892	0.0002130833156	1
upheno	0.001491583209	0.000002435237892	0.0002130833156	1
wbls	0.001491583209	0	0	1
cob	0.001491583209	0	0	1
ppo	0.001278499893	0.08606065772	0.003409333049	1
bspo	0.001278499893	0.0001392144328	0.001491583209	1
orth	0.001278499893	0.00001775694296	0.0005327082889	1
tads	0.001278499893	0.0000113644435	0.0004261666312	1
clyh	0.001278499893	0.000002841110874	0.0002130833156	1
vario	0.001278499893	0.000002841110874	0.0002130833156	1
zfs	0.001278499893	0.000002841110874	0.0002130833156	1
caro	0.001278499893	0	0	1
aeo	0.001065416578	0.05179177498	0.001598124867	1
tto	0.001065416578	0.0001670573194	0.001491583209	1
cmpo	0.001065416578	0.0001227359898	0.001278499893	1
mop	0.001065416578	0.00008523332623	0.001065416578	1
labo	0.001065416578	0.00002130833156	0.0005327082889	1
trans	0.001065416578	0.000003409333049	0.0002130833156	1
amphx	0.001065416578	0.000003409333049	0.0002130833156	1

hsapdv	0.001065416578	0	0	1
olatdv	0.001065416578	0	0	1
ddpheno	0.001065416578	0	0	1
gno	0.0008523332623	0	0	1
fbdv	0.0008523332623	0	0	1
pdumdv	0.0008523332623	0	0	1
wbbt	0.0008523332623	0	0	1
poro	0.0006392499467	0.0001150649904	0.0009588749201	1
colao	0.0006392499467	0.00001278499893	0.0003196249734	1
mro	0.0006392499467	0	0	1
msio	0.0006392499467	0	0	1
prov	0.0006392499467	0	0	1
mmusdv	0.0006392499467	0	0	1
ensemblglossary	0.0004261666312	0.0003601108033	0.001385041551	1
hancestro	0.0004261666312	0.01900852333	0.0002130833156	1
cteno	0.0004261666312	0	0	1
cheminf	0.0002130833156	0	0	1
kisao	0.0002130833156	0	0	1

Table 1: Results of ontology evaluation and selection process, ordered by descending SetCoverage.
