# Supplementary material for: Processing genome-wide association studies within a repository of heterogeneous genomic datasets
Source: BMC Genom Data. 2023 Mar 3;24:13. doi: 10.1186/s12863-023-01111-y (PMC9985298; doi:10.1186/s12863-023-01111-y)
Supplement: Supplementary file 5 — Additional file 5. Common traits and mapping to ontologies. [file 12863_2023_1111_MOESM5_ESM.pdf]

Additional File 5

Anna Bernasconi[1], Arif Canakoglu[1], and Federico Comolli[1]

[1]Dept. of Electronics, Information and Bioengineering (DEIB), Politecnico di Milano, 20133 Milano, Italy

Common traits and mapping to ontologies

Table 1: Traits from FinnGen and GWAS Catalog mapped to the same terms of EFO or NCIT. A few entries present ontology ids from other ontologies, but these are also contained in either EFO or NCIT.

**Ontology ID**	**List of traits mapped to the ID from the two sources**
EFO_0000274	finngen: atopic dermatitis, gwas: atopic eczema, gwas: atopic eczema, covid-19
EFO_0000712	finngen: accidents, finngen: stroke, finngen: stroke, excluding sah, finngen: stroke, including sah, gwas: stroke
EFO_0000729	finngen: other ulcerative colitis, gwas: ulcerative colitis, crohn's disease
EFO_0001062	finngen: cytomegaloviral disease, gwas: schizophrenia, cytomegalovirus infection
EFO_0003819	finngen: dental caries, gwas: dental caries, gwas: dental caries, dentures
EFO_0003948	finngen: gastro-oesophageal reflux disease, gwas: esophageal adenocarcinoma, gastroesophageal reflux disease
EFO_0004191	finngen: androgenic alopecia, gwas: androgenetic alopecia
EFO_0004257	finngen: other neurotic disorders, gwas: neurotic disorder
EFO_0004265	finngen: peripheral artery disease, gwas: peripheral arterial disease
EFO_0004683	finngen: wet age-related macular degeneration, gwas: wet macular degeneration
EFO_0004772	finngen: early onset copd, gwas: early onset hypertension
EFO_0006812	finngen: autoimmune thyroiditis, gwas: autoimmune thyroid disease
EFO_0007135	finngen: adult-onset still disease, gwas: adult-onset still's disease
EFO_0009458	finngen: alcohol use disorder, icd-based, finngen: alcohol use disorder, swedish definition, gwas: alcohol use disorder measurement, gwas: alcohol use disorder measurement, alcohol dependence
EFO_0010822	finngen: trigger finger, gwas: stenosing tenosynovitis
EFO_1000786	finngen: coxarthrosis,, gwas: osteoarthritis, hip, gwas: osteoarthritis, hip, osteoarthritis, knee
EFO_1000941	finngen: adhesive capsulitis of shoulder, gwas: frozen shoulder
EFO_1000965	finngen: allergic purpura, gwas: henoch-schoenlein purpura
EFO_1001178	finngen: impingement syndrome of shoulder, gwas: shoulder impingement syndrome
EFO_1001250	finngen: rotator cuff syndrome, gwas: rotator cuff tear
EFO_1001436	finngen: nontoxic single thyroid nodule, gwas: thyroid nodule
EFO_1001494	finngen: psoriasis vulgaris, finngen: psoriasis (vulgaris), strict definition, gwas: psoriasis vulgaris
EFO_1001506	finngen: primary angle-closure glaucoma, gwas: primary angle closure glaucoma
EFO_1001507	finngen: acohol-induced acute pancreatitis, gwas: asparaginase-induced acute pancreatitis
HP_0000708	finngen: behavioural disorders, gwas: behavioral abnormality
HP_0100806	finngen: puerperal sepsis, gwas: sepsis
MONDO_0005405	finngen: childhood asthma (age¡16), gwas: childhood onset asthma
NCIT_C114357	finngen: other juvenile arthritis, gwas: juvenile idiopathic arthritis
NCIT_C117004	finngen: migraine without aura, finngen: migraine without aura, drug-induced, gwas: migraine without aura
NCIT_C117005	finngen: migraine with aura, finngen: migraine with aura, drug-induced, gwas: migraine with aura
NCIT_C118764	finngen: amblyopia ex anopsia, gwas: amblyopia
NCIT_C122822	finngen: cholelithiasis, gwas: cholelithiasis, gallstones
NCIT_C142891	finngen: cr(e)st syndrome, gwas: brugada syndrome
NCIT_C157552	finngen: hypertension, pulmonary arterial, gwas: pulmonary arterial hypertension
NCIT_C164315	finngen: interstitial lung disease, finngen: interstitial lung disease endpoints, gwas: interstitial lung disease
NCIT_C26696	finngen: all anxiety disorders, finngen: phobic anxiety disorders, gwas: anxiety
NCIT_C26713	finngen: other cataract, gwas: cataract
NCIT_C26748	finngen: diabetic neuropathy, gwas: diabetic foot, neuropathy, gwas: diabetic neuropathy
NCIT_C26782	finngen: glaucoma, finngen: glaucoma suspect, finngen: normotensive glaucoma, gwas: glaucoma
NCIT_C26800	finngen: hypothyroidism, levothyroxin purchases, finngen: hypothyroidism, other/unspecified, finngen: hypothyroidism, strict autoimmune, finngen: postinfectious hypothyroidism, gwas: hypothyroidism
NCIT_C26845	finngen: parkinson's disease, finngen: parkinson's disease, including avohilmo, finngen: parkinson's disease, strict definition, gwas: parkinson's disease
NCIT_C27195	finngen: acne, finngen: acne vulgaris, gwas: acne
NCIT_C27996	finngen: complications following myocardial infarction, finngen: myocardial infarction, finngen: myocardial infarction, strict, gwas: myocardial infarction
NCIT_C28397	finngen: asthma, finngen: asthma, including avohilmo, gwas: allergy, asthma, gwas: asthma
NCIT_C2884	finngen: other/unspecified rheumatoid arthritis, finngen: rheumatoid arthritis, finngen: seronegative rheumatoid arthritis, finngen: seropositive rheumatoid arthritis, finngen: seropositive rheumatoid arthritis, wide, gwas: rheumatoid arthritis, gwas: rheumatoid arthritis, follicular lymphoma
NCIT_C2952	finngen: ulcerative colitis, finngen: ulcerative colitis, nas, gwas: rheumatoid arthritis, ulcerative colitis, gwas: ulcerative colitis
NCIT_C2985	finngen: diabetes mellitus, finngen: diabetes, varying definitions, gwas: diabetes mellitus
NCIT_C3001	finngen: dermatitis and eczema, gwas: eczema
NCIT_C3123	finngen: autoimmune hyperthyroidism, gwas: hyperthyroidism
NCIT_C3163	finngen: chronic lymphocytic leukaemia, gwas: chronic lymphocytic leukemia, gwas: multiple sclerosis, chronic lymphocytic leukemia, gwas: rheumatoid arthritis, chronic lymphocytic leukemia
NCIT_C3199	finngen: copd, finngen: other chronic obstructive pulmonary disease, gwas: chronic obstructive pulmonary disease, gwas: chronic obstructive pulmonary disease, asthma, gwas: hypertension, chronic obstructive pulmonary disease
NCIT_C3201	finngen: systemic lupus erythematosus, unspecified, gwas: rheumatoid arthritis, systemic lupus erythematosus, gwas: systemic scleroderma, systemic lupus erythematosus
NCIT_C3209	finngen: follicular lymphoma, finngen: non-follicular lymphoma, gwas: multiple sclerosis, follicular lymphoma
NCIT_C3243	finngen: ms-disease / multiple sclerosis, gwas: multiple sclerosis, gwas: multiple sclerosis, disease recurrence, gwas: multiple sclerosis, triglyceride measurement
NCIT_C3256	finngen: nasal polyp, gwas: nasal cavity polyp
NCIT_C3298	finngen: osteoporosis, gwas: obesity, osteoporosis, gwas: osteoporosis
NCIT_C3303	finngen: pain in joint, finngen: tmd related pain, gwas: pain
NCIT_C3423	finngen: respiratory tuberculosis, finngen: respiratry tuberculosis, finngen: tuberculosis, gwas: tuberculosis
NCIT_C3444	finngen: wegener granulomatosis, gwas: granulomatosis with polyangiitis
NCIT_C34440	finngen: bulimia nervosa (incl. atypical), gwas: bulimia nervosa
NCIT_C34525	finngen: vascular dementia, finngen: vascular dementia (mixed), finngen: vascular dementia (undefined), gwas: vascular dementia
NCIT_C34538	finngen: diabetic retinopathy, finngen: other diabetic retinopathy, finngen: unspecified diabetic retinopathy, gwas: diabetic retinopathy
NCIT_C34549	finngen: disturbances of skin sensation, gwas: disturbance of skin sensation
NCIT_C34650	finngen: gout, finngen: gout, finngen, finngen: gout, strict definition, finngen: gout, unspecified, finngen: idiopathic gout, gwas: gout
NCIT_C34658	finngen: hallux valgus (acquired), gwas: hallux valgus
NCIT_C34690	finngen: inguinal hernia, finngen: inguinal hernia, bilateral, finngen: inguinal hernia, unilateralk, gwas: inguinal hernia
NCIT_C34882	finngen: rheumatic fever incl heart disease, gwas: rheumatic heart disease
NCIT_C34885	finngen: otitis media, unspecified, gwas: otitis media
NCIT_C34890	finngen: panic disorder, gwas: anxiety disorder, panic disorder, gwas: panic disorder
NCIT_C34942	finngen: gestational diabetes (for exclusion), gwas: gestational diabetes
NCIT_C35040	finngen: other strabismus, finngen: strabismus, finngen: vertical strabismus, gwas: strabismus
NCIT_C35065	finngen: giant cell arteritis, finngen: temporal arteritis, gwas: temporal arteritis
NCIT_C3510	finngen: malignant melanoma of skin, gwas: cutaneous melanoma, gwas: neuroblastoma, cutaneous melanoma
NCIT_C35145	finngen: acute appendicitis, finngen: appendicitis, broad definition, finngen: other appendicitis, gwas: appendicitis
NCIT_C35731	finngen: other hearing loss, gwas: hearing loss
NCIT_C35768	finngen: peripheral atherosclerosis, gwas: atherosclerosis
NCIT_C38012	finngen: isolated proteinuria, gwas: proteinuria
NCIT_C4731	finngen: diabethic neuropathy, finngen: intercostal neuropathy, gwas: neuropathy
NCIT_C50577	finngen: all-cause heart failure, finngen: heart failure, not strict, finngen: heart failure,strict, gwas: heart failure
NCIT_C61277	finngen: psoriatic arthropathies, gwas: psoriatic arthritis
NCIT_C63709	finngen: temporomandibular joint disorders, gwas: temporomandibular joint disorder
NCIT_C72070	finngen: systemic sclerosis, gwas: systemic scleroderma, gwas: systemic scleroderma, crohn's disease
NCIT_C79532	finngen: allergic rhinitis, gwas: allergic rhinitis, gwas: eczema, allergic rhinitis, gwas: eczema, allergic rhinitis, asthma
NCIT_C80078	finngen: chronic kidney disease, gwas: chronic kidney disease, gwas: chronic kidney disease, diabetic nephropathy, gwas: creatinine measurement, chronic kidney disease
NCIT_C84417	finngen: diabetic nephropathy, gwas: albuminuria, diabetic nephropathy, gwas: diabetic nephropathy
NCIT_C84564	finngen: ankylosing spondylitis, finngen: ankylosing spondylitis, strict definition, gwas: ankylosing spondylitis
NCIT_C8851	finngen: diffuse large b-cell lymphoma, gwas: diffuse large b-cell lymphoma, gwas: diffuse large b-cell lymphoma, rheumatoid arthritis
NCIT_C94378	finngen: schizoaffective disorder, gwas: schizophrenia, schizoaffective disorder
NCIT_C94569	finngen: tooth eruption problems, gwas: tooth eruption
NCIT_C97161	finngen: autism, finngen: autism spe, gwas: autism
